# Characterization of bovine (*Bos taurus*) imprinted genes from genomic to amino acid attributes by data mining approaches

**DOI:** 10.1371/journal.pone.0217813

**Published:** 2019-06-06

**Authors:** Keyvan Karami, Saeed Zerehdaran, Ali Javadmanesh, Mohammad Mahdi Shariati, Hossein Fallahi

**Affiliations:** 1 Department of Animal Science, Faculty of Agriculture, Ferdowsi University of Mashhad, Mashhad, Iran; 2 Department of Biology, School of Sciences, Razi University, Kermanshah, Iran; Chang Gung University, TAIWAN

## Abstract

Genomic imprinting results in monoallelic expression of genes in mammals and flowering plants. Understanding the function of imprinted genes improves our knowledge of the regulatory processes in the genome. In this study, we have employed classification and clustering algorithms with attribute weighting to specify the unique attributes of both imprinted (monoallelic) and biallelic expressed genes. We have obtained characteristics of 22 known monoallelically expressed (imprinted) and 8 biallelic expressed genes that have been experimentally validated alongside 208 randomly selected genes in bovine (*Bos taurus*). Attribute weighting methods and various supervised and unsupervised algorithms in machine learning were applied. Unique characteristics were discovered and used to distinguish mono and biallelic expressed genes from each other in bovine. To obtain the accuracy of classification, 10-fold cross-validation with concerning each combination of attribute weighting (feature selection) and machine learning algorithms, was used. Our approach was able to accurately predict mono and biallelic genes using the genomics and proteomics attributes.

## Introduction

Most diploid organisms, including mammalians, receive two copies of each gene from their parents and express both alleles equally in their cells. For normal development, each individual needs to receive both the maternal and paternal genomes. For many genes in mammalian species, both the maternal and paternal alleles are equally expressed. However, the expression of some genes is determined by imprinting, an epigenetic event in which only one of the alleles inherited from one of the parents get silenced and inactivated[1]. Consequently, in a limited group of genes which are imprinted, one of the parental alleles is expressed preferentially [2]. The epigenetic mechanism in the form of imprinting leads to monoallelic expression of some genes depending on parent-of-origin of the allele [3]. Therefore, if the paternal allele of the gene is imprinted, the other allele from the mother would be expressed and vice versa. This results in unequal expression of two alleles of a gene, which is in contrast to Mendelian genetics. The imprinting mechanism in mammalian species is mainly conserved. Genomic imprinting leads to allele-specific gene expression[4, 5]. It has been shown that many human diseases including Prader–Willi syndrome (PWS)[6], Beckwith–Wiedemann syndrome (BWS) [7] and some types of cancer [8, 9] are strongly associated with defective expression in imprinted genes. Large offspring syndrome (LOS) is an example of abnormal imprinting in bovine and ovine that causes abnormally high rates of growth which is phenotypically and epigenetically similar to BWS in human [10]. The conservation pattern between different organisms has greatly facilitated the study of imprinting mechanisms in some human genetic disorders [11]. On the other hand the importance of imprinted genes is increasing, because there are some evidences that imprinting defects are associated with complex disorders like diabetes, obesity, developmental abnormalities and behavioral disorders.

Although single imprinted genes could be observed in the genome, imprinted genes typically located in clusters with 3 to 12 genes with a length of 20 kb to 3.7 Mb of DNA[11].The clustering of imprinted genes is the key mark that the imprinting process is not specific to the gene and can act through cis orientation with elements controlling the expression of multiple genes. Experiments identified key controllers as imprint control element (ICE) or imprint control region (ICR) in seven imprinted clusters. It has been showed that deleting this element in mouse leading to loss of imprinted expression following transmission through the maternal or paternal germline [12]. In fact, when ICE is un-methylated, it operates as an initiator which epigenetically repress genes in a cis position manner [13]. Although DNA methylation, histone modifications and non-coding RNAs (ncRNAs) are key mechanisms in genomic imprinting, the genomic sequence is still important [14].

Most of imprinted genes play important roles in the growth regulation of fetus and placenta in the process of development [15, 16]. Many imprinted genes have been identified in human and mouse while few imprinted genes have been identified in cattle, might indicate the limitations of the bovine data set. Computational prediction of imprinted genes using attributes in the genomic DNA sequences alone has been used for mouse and human genomes [17, 18]. It has been reported that imprinted genes in mammals include only one percent of the whole genome. However, a wide range of imprinted genes from 100 [18] to 600 genes [17] and even more than 2000 genes [19] is reported in the literature. The variation may be due to ignorance of tissue-specific and conditional imprinting statues for some of imprinted genes. Interestingly, there are many genes that are imprinted in human or mouse but not imprinted in cattle[3]. Although the genome of bovine with the highest percentage of annotated genes and a high sequence coverage is the most characterized genome among livestock, few imprinted genes have been experimentally identified [20]. At present, only 25 imprinted genes experimentally validated in cattle and this list could be expanded by adding new imprinted genes [21, 22]. With a complete catalog of imprinted genes in different mammalian species, it is feasible to understand the role of genomic imprinting in the evolutionary process [23]. With this quantity of confirmed imprinted genes in the bovine, there is a chance to find other genomic characteristics common to bovine imprinted genes.

In this study, several sequence attributes were selected to find common characteristics shared among imprinted genes. Because of known association of differential methylation with imprinting status in this region, GC contents and CpG islands were tested. It has been suggested that high-density CpG islands, tandem repeat patterns, and retrotransposons were selective characteristics of imprinted Differentially Methylated Regions (DMRs) [24]. Gene expression, histone modifications and transcription factor (TF) binding sites are strongly correlated with methylation mechanism in DNA [25]. The epigenetic regulation through methylation of CpG islands is an important mechanism in the differentiation of embryonic stem cells into specific cells and tissues [24]. Attributes of 20 known imprinted genes in human, mouse, and cattle species were previously described and compared by Khatib et al. (2007). They observed higher values for GC contents, CpG islands and tandem repeats in imprinted genes. In several imprinted genes, DMRs were related to monoallelic gene expression pattern [3]. DNA methylation occurs largely in repetitive elements including satellite sequences, centromeric repeats, and CpG islands in or near promoter sequences. CpG islands are GC rich areas in DNA with high proportions of CpG dinucleotides.

According to the original definition, CpG islands is defined as a region with GC content greater than 50%, at least 200 bp length and CpGs observed to expected ratio above 0.6 [26]. CpG islands are commonly regarded as epigenetic key regulatory elements. Although CpG islands are generally un-methylated, a significant number of CpG islands are methylated in genes promoter regions. Methylation in the promoter region is generally related to the silencing of genes related to different types of cancers [27]. Thus, gene silencing can generally occur after hyper-methylation of CpG islands in the promoter region which leads to inactive transcription. The level of DNA methylation has also been found to be related to gene length [28]. Other studies showed that retrotransposons or tandem repeats are unconfidently predictors of imprinting statues [29, 30]. Repetitive elements are nucleotide patterns that, in contrast to unique sequences, occur multiple times in the genome. In fact, a substantial part of the mammalian genome consists of repeats. In accordance with previous reports in human and mouse, imprinted genes in cattle have remarkably fewer Short Interspersed Elements (SINEs) in number compared to biallelic expressed genes. Also, imprinted genes were found significantly underrepresented Long Interspersed Elements (LINEs) and Long Terminal Repeats (LTRs) in compare to biallelic expressed genes, which is in contrast with studies on human and mouse. Cowley et al. (2011) by observing no differences in repeat element prevalence at imprinted retrogene loci, concluded that the SINE depletion and LINE abundance were not required features for imprinting. Several studies reported differences between imprinted loci and biallelic expressed genes in the occurrence of the repeated sequences biallelic. A general agreement is that SINEs to be depleted at imprinted loci with increased frequency of LINEs. By taking the high density of LINE-1 elements around X-inactivated genes into account, it can be confirmed that the monoallelic expression of autosomal genes is flanked by high densities of repetitive sequences [31].

Additionally, some studies suggested a correlation between codon usage and the level of gene expression [32, 33]. Therefore, in the current study codon usage by mono and biallelic genes was considered as a feature. To our knowledge, this is the first study that amino acid and codon usage attributes were used for determining characteristics of imprinted and biallelic expressed genes.

The purpose of machine learning methods is learning functional relationships from data without defining a priori [34–36]. In computational biology, the purpose is to obtain predictive models without strong assumptions about underlying mechanisms, which are less well known or unknown [37]. Since bioinformatics was introduced, researchers used machine learning to accelerate studies on biomolecular structure prediction, gene finding, genomics and proteomics [38]. We have hypothesized that data mining approaches would find unique attributes of mono and biallelic genes. Classification algorithms try to build a classification model given some examples of the classes we are trying to model. The obtained model can then be used to improve our knowledge on available data. The performance of a machine learning algorithm for classification tasks can be intensively influenced by the relevance of attributes. This performance can be easily diminished if redundant attributes are used [39]. Attribute weighting models create a set of more relevant attributes by reducing their size [40]. In some functional examples, different supervised and unsupervised machine learning algorithms were used by Ebrahimi et al. (2011) to capture attributes that related to thermostability of proteins [41]. Beiki et al. (2012) also used different supervised and unsupervised machine learning algorithms to classify and predict of olive cultivars [42]. Hosseinzadeh et al. (2012) used machine learning approaches to classify different types of lung cancer [43]. Clustering is a grouping process so that the objects in each cluster have high similarity with each other but they have high dissimilarity with objects in other clusters [44]. Discovering the relationship between input attributes and a target attribute is the main characteristics of supervised methods. The discovered relationship is then used in the structure of the model. Discovering structure by exploring similarities or differences among individual data is the ability of a relevant unsupervised algorithm [45]. Clustering is considered to be the most important problem of unsupervised learning method. Meanwhile, with little or no knowledge, relevant patterns and structures can be found directly from datasets by clustering [46]. Clustering problems can be solved easily by K-Means [47], which is a simple unsupervised learning algorithm. The process uses a simple method to classify a given dataset through a certain number of clusters (assumed k clusters) fixed a priori. Representative objects instead of the mean value of objects in each cluster are used by the K-Medoids methods as reference points.

The aim of the present study was to identify unique characteristics to distinguish mono from biallelic expressed genes in the bovine genome using supervised and unsupervised machine learning algorithms and attribute weighting methods.

## Materials and methods

### Dataset

A list of 30 genes containing 22 verified imprinted and 8 biallelic expressed genes in the bovine were compiled from the MetaImprint (http://bioinfo.hrbmu.edu.cn/MetaImprint) and GeneImprint database (http://www.geneimprint.com). The list consisted of *GRB10*, *PEG10*, *SGCE*, *MEST*, *NAP1L5*, *IGF2R*, *MAOA*, *GNAS*, *PEG3*, *NDN*, *MEG3*, *RTL1*, *APEG3*, *NNAT*, *TSSC4*, *H19*, *IGF2*, *PHLDA2*, *CDKN1C*, *MEG9*, *PLAGL1 and ASCL2* as experimentally validated imprinted genes and *COPG2*, *ASB4*, *SLC38A4*, *HTR2A*, *SFMBT2*, *SDHD*, *CD81 and DCN* as biallelic expressed genes. There are conflicting reports surrounding the status of *SNRPN* and *ZIM2* genes to be mono or biallelic expressed genes. Therefore, we did not use these genes in our analysis. Furthermore, *XIST* gene is also a particularly challenging example due to its pattern of expression; it is randomly expressed in rodent, human and embryonic tissues [48], while it is just expressed paternally in the rodent extraembryonic tissues [49]. As such, *XIST* gene was also not included in our analysis. In spite of the fact that almost majority of genes in the genome have biallelic expression patterns, low number of validated non-imprinted genes were available in the bovine genome. Therefore, we randomly selected 200 genes from bovine genome to increase the number of genes in biallelic dataset. Therefore, with 8 validated non-imprinted genes and 200 randomly selected genes, initial dataset comprised 208 non-imprinted and 22 imprinted genes (S1 Table).

### Extracted attributes from genomic and protein sequences

The initial dataset contained 200 attributes in three levels containing DNA structure, transcription level as codon usage and amino acids composition. CpG islands, SINEs, LINEs, LTRs, described for the DNA sequence, were extracted from the UCSC Genome Browser website (http://genome.ucsc.edu/cgi-bin/hgGateway; February 2006 build), using table tools. UCSC Repeat Masker tracks were used for finding of repeated elements. Also, GC contents were analyzed using the R environment (http://www.r-project.org) via BS genome package (BSgenome.Btaurus.bosTau8). The Uni-Prot Knowledgebase (Swiss-Prot and Tremble) database was used for extracting protein sequences. A number of attributes such as count and frequency of each amino acid and negatively and positively charged amino acids were extracted using various bioinformatics tools and softwares from the ExPASY website (http://www.expasy.org). Codon usages of genes shown by the frequency of each codon types were obtained from www.bioinformatics.org/sms2/codon_usage.html.

Attributes were individually extracted for 11 genomic sub-domains. These attributes consisted of genes as whole, exons, introns, 5'-untranslated region (5'-UTR), 3'-untranslated region (3'-UTR), +1, +10, +100 kb upstream, -1, -10, and -100 kb downstream. Consequently, genomic coordinates for total numbers of SINEs, LINEs, LTRs and simple repeats in genes sequence length, exons, introns, 5'-UTR, 3'-UTR, +1 kb, +10 kb, +100 kb, -1 kb, -10 kb, and -100 kb regions of both mono and biallelic expressed genes were calculated. Additionally, number of CpG islands (CpGi), number of CpG dinucleotide (CpGn) and CpGi length, in these regions were assessed. Number of SINEs, LINEs, LTRs, simple repeats, CpGi and CpGn were also calculated per kb of gene regions sequence (from start to end). In the cases of ambiguous attributes, such as loss of intron in some genes, the missing values filled by average attribute values.

The dataset was imported into the RapidMiner (RapidMiner7.5.003, www.rapidminer.com), and imprinted and biallelic expressed genes were set as the target or label attribute. In addition, differences between imprinted and biallelic expressed genes regarding attributes were statistically tested using Mann-Whitney U test. Distributions of these attributes across imprinted and biallelic expressed genes were then visualized as boxplot graphs using BoxPlotR web tools[50].

### Data cleaning

To eliminate repeating characteristics, attributes with correlation coefficient higher than 0.95 were not considered. Furthermore, numerical attributes with standard deviation lower than or equal to 0.1 were removed. The final refined dataset was considered as the main source and labeled as Mds dataset.

### Attribute weighting

Attribute weighting is a method of choice to identify attributes contributing to objects. This method was used to identify important attributes and their contribution to allele-specific expression. The procedure suggested by Ebrahimi et al. (2011) was used as the main guide to do attribute weighting. Eleven attribute weighting algorithms consisting weight by information gain, weight by information gain ratio, weight by principle component analysis, weight by correlation, weight by rule, weight deviation, weight by chi squared statistic, weight by Gini index, weight by uncertainty, weight by relief, weight by support vector machine were used in the main dataset.

In weight by information gain algorithm, the relevance of each attribute was evaluated by computing the information gain in class distribution. Higher weight for an attribute means it is more appropriate than others. In weight by information gain ratio, the information gain ratio for the class distribution is an indicator of the relevance of each attribute. Weight by principle component analysis (PCA) operator creates attribute weights using a component generated by the PCA. In PCA an orthogonal transformation is used to convert values of correlated attributes into observations of uncorrelated attributes. The weight of each attribute shows its importance with respect to the class attribute. In weight by correlation operator, the weight of each attribute with respect to the label attribute is calculated using correlation. The weight of each attribute show its relevance. In weight by Rule operator, the importance of each attribute of the given example set is identified by constructing a single rule for each attribute and estimating errors. In weight deviation operator, weights are created from the standard deviation of all attributes. Normalization of values is done by the average, minimum, or maximum of the attribute. In weight by chi-square statistic, the relevance of an attribute was computed based on the value of the chi-square statistic with respect to the class attribute. The chi squared statistic is used to find out whether a distribution of observed frequencies differs from expected frequencies. In chi-squared statistics, frequencies are used instead of means and variances. Weight by Gini index is another operator that identify relevant attributes by calculating the Gini index of the class distribution. In weight by uncertainty operator, the relevance of an attribute was identified by calculating the symmetrical uncertainty with respect to the class. Weight by Relief is another operator which measures the relevance of attributes by sampling examples and comparing the value of the current attribute for the nearest example of the same and of a different class. Obtained weights are normalized into the range of 0 and 1. Weight by Support Vector Machine (SVM) is an operator that attribute weights are coefficients of the normal vector of a linear SVM [51].

### Selection of Attributes

A value between 0 and 1 was obtained for each attribute, after performing attribute weighting models on the Mds. This value shows the relevance of the attribute with regards to the imprinted or biallelic expressed gene as a target attribute. Variables were selected with weights more than 0.50 and consequently 11 new datasets were created (Awds) (Table 1). These data sets were named based on their attribute weighting models (Info Gain, Info Gain Ratio, PCA, Correlation, Rule, Deviation, chi squared, Gini index uncertainty, relief and SVM) and then supervised and unsupervised models were used. Each model of the supervised or unsupervised algorithm was performed 12 times, first on the Mds and then on the new eleven datasets (Awds).

**Table 1:** Attributes selected by different algorithms of attribute weightings.

**Chi-squared statistic**	CpGn 3UTR, Ala, Arg, CpGi introns, CpGn 5UTR, CpGn introns, SINE 100kbUp, Avg CpG 100kbUp length, Pro, GC 10kbdwn, Avg CpG 5UTR length
**Information gain**	Ala, CpGn 3UTR, Avg CpG 3UTR length, Pro, SINE 100kbUp, Pro/CCT, Arg, Avg CpG 100kbDwn length, Ile, Lys, SINE 10kbUp, CpGn introns, Avg CpG 5UTR length, CpG 100kbDwn length, N amino aicds, CpG 100kbUp length, Avg CpG 100kbUp length, His/CAC, CpGi gene/kb, Pro/CCG, CpGn gene/kb, CpGn 5UTR, CpGi introns, LINE Introns, SINE Introns, Avg CpG introns length, simplrep/kb Gene, GC introns, Asn
**Deviation**	CpG 100kbUp length, length first intron
**Gini index**	Ala, CpGn 3UTR, Pro, Pro/CCT, CpGn introns, SINE 100kbUp, Avg CpG gene length, His/CAC, CpGi introns, N amino aicds, Arg, GC 10kbup, CpGn gene, Avg CpG 5UTR length, CpGn gene/kb, Pro/CCG, CpGi gene/kb, SINE 10kbUp, Avg CpG 100kbDwn length, SINE Introns, Avg CpG 100kbUp length, simplrep/kb Gene
**Information Gain ratio**	CpGn gene, GC 10kbup, CpGn 5UTR, Ala, GC first exone, GC introns, GC 3UTR, GC 5UTR, GC 10kbdwn, GC 1kbup, length first exone, CpGi gene, AvgCpG5’-UTR length, CpGiexnos, CpGi introns, AvgCpG 10kbUp length, CpGi 100kbUp, SINE 100kbUp, LINE 100kbUp, simplrep 100kbUp, Arg, Gly, His, Thr, Ncharg (Asp+Glu), NchargeFreq, Pcharge (Arg+Lys), N amino aicds, CpGn3’-UTR
**Principle Component Analysis**	CpG 100kbUp length
**Correlation**	Ala, CpGn gene, CpGn 3UTR, SINE 10kbUp, CpGn 5UTR, Avg CpG 5UTR length, CpGi introns, SINE 100kbUp, GC introns, His/CAC, Avg CpG gene length, Ile, CpGn introns, Pro/CCG, CpGi gene, CpGi 100kbUp, Arg, GC 1kbup, Avg CpG 100kbDwn length, Pro, simplrep/kb Gene, Lys, SINE 10kbDwn, GC genes, CpGi exnos, GC 10kbdwn, N amino acids, Leu/CTG, Pro/CCT, GC 1kbdwn, SINE 1kbUp, Ncharg (Asp+Glu), Avg CpG exons length, length first exone, GC 100kbdwn, GC exons, Pcharge (Arg+Lys), CysTGC, Arg/AGA
**Relief**	GC 100kbdwn, GC 10kbdwn, CpGi 100kbDwn, CpGi 1kbDwn, GC 100kbup, GC 10kbup, GC introns, CpGn 100kbUp, GC 1kbdwn, Avg CpG 100kbUp length, CpGi 1kbUp, Ala, Avg CpG 5UTR length, CpGi 100kbUp, CpGn 10kbUp, GC genes, CpGi 10kbDwn, SINE 100kbUp, CpGn 100kbDwn, LINE 100kbUp, Tyr/TAC, simplrep 100kb Dwn, Tyr/TAT
**Rule**	GC 3UTR, GC 5UTR, GC 10kbdwn, GC 1kbup, Ncharge Freq, GC first exone, GC 10kbup, Avg CpG 10kbUp length, GC introns, length first exone, Avg CpG 5UTR length, N amino aicds, Ala, Arg, CpGn gene, Ncharg (Asp+Glu)
**Uncertainty**	CpGn 3UTR, CpGi introns, CpGn introns
**SVM (Support Vector Machine)**	CpG 100kbDwn length, Avg CpG 100kbDwn length, SINE 100kbUp, CpGi 3UTR, Ala, Avg CpG gene length, SINE 10kbUp, CpGn 3UTR, Avg CpG 10kbDwn length, CpGn gene, CpGn 1kbDwn, Pro/CCT

Seqlength = length of sequence, CpGi = CpG island, CpGi gene/kb = CpG island per kb of gene region, CpGn = number of CpG dinucleotide, CpGn gene/kb = number of CpG dinucleotide per kb of gene region, CpGn exons = number of CpG dinucleotide in exon region, LINE/kb Gene = number of LINE elements per kb gene region, SINE/kb Gene = number of SINE elements per kb gene region, LINE+SINE+LTR /kb Gene = number of LINE and SINE and LTR per kb gene region, 1–10–100 kb UP and Dwn = 1, 10, 100 kb up and down stream, simplrepExones = simple repeat in exon region.

### Supervised classification

Finding the correlation between input and target attributes was the main aim of supervised methods. The supervised classification was applied on the main dataset and the eleven newly created datasets from attribute weighting.

#### Decision trees

Four tree models i.e. decision tree, decision stump, random tree, and random forest were performed on Mds and Awds.

#### Neural network and Bayesian models

In quantitative modeling, the artificial neural network is an important analysis tool. Pattern classification, time series analysis, and prediction and clustering are examples of data mining tasks that can be done by neural network [52]. Four neural network models consisting, deep learning, neural net, autoMPL, and perceptron were performed on all datasets. Nowadays, deep learning is considered the highest usage fields in machine learning in computational biology [53–60]. Deep Learning was trained with stochastic gradient descent and in this process, back-propagation was used. Neural network learns a model by a feed-forward neural network trained using back-propagation algorithm (multi-layer perceptron). The feed-forward neural network or multilayer perceptron (MLP), are widely used neural network models in practice [52]. AutoMLP is an algorithm which can be used for learning rate and adjustment size of neural networks during training. The perceptron is the simplest kind of feed-forward neural network. The single layer perceptron is a linear classifier efficiently trained by a simple update rule for all classified data points.

Three Bayesian models consisting Naive Bayse, Bayse Kernel, and W-BayesNet were performed on all dataset. A Naive Bayes classifier is a simple probabilistic classifier based on Bayes' theory with strong independence assumptions. In other words, a Naive Bayes classifier assumes that a specific attribute is not related to any other attributes. Naive Bayes is a popular machine learning algorithm performing well in many domains, and mostly used in binary sentiment classification [61–63]. The Naive Bayes (Kernel) operator can be used for numerical attributes which are in contrast with the Naive Bayes operator. The kernel is a non-parametric estimation technique that is used as a weighting function.

### Unsupervised clustering

Unsupervised learning involves methods that group instances with any kinds of pre-specification. The unsupervised clustering was applied on the main dataset and the eleven generated data sets from attribute weighting.

### K-means and k-medoids

K-means operator predicts the distance of objects to clusters using kernels. Considering the property of kernels, for calculating one distance all elements of a cluster must be generally added. K-means is known as one of the simplest algorithms of the parametric unsupervised technique [46]. K-medoids algorithm is a partitioned clustering algorithm which is slightly modified from the K-means algorithm. They both attempt to minimize the squared-error but the K-medoids algorithm is more robust to noise than K-means algorithm. In K-means algorithm, means are chosen as the centroids but in the K-medoids, data points are chosen to be the medoids. A medoid can be defined as that object of a cluster, whose average dissimilarity to all the objects in the cluster is minimal.

### Accuracy of models

To identify the performed classification and to estimate the accuracy of using each attribute,10-fold cross validation (10-fold CV) with stratified sampling was applied to train and test models on all patterns. Some random subsets are generated by stratified sampling in order to make sure that there is no difference between class distribution in the subsets and in the whole example set and almost the same rate of two values of class labels were in each subset. To do this, 10 parts were generated from all records randomly, the training phase was performed on 9 parts and testing phase was done on the 10^th^ part. The accuracy for true and false and total accuracy was calculated after repeating the process for ten times. The mean of the accuracy for all ten tests was reported as the final accuracy. The same cross-validation was used for all the evaluation methods and each method had the same training and testing sets used during the genetic algorithms run.

## Results

### Data cleaning

The initial dataset contained 230 imprinted (monoallelic) and biallelic expressed genes with 200 attributes. 22 genes have been experimentally validated as imprinted genes and 208 genes were biallelic. After taking into account the standard deviation of each attribute and Pearson correlation coefficients between them, the amount of attributed used in the current study decreased to 164 more informative non-redundant attributes (Mds).

### Attribute selection via attribute weighting

A list of 20 most important attributes selected by algorithms of attribute weighting has been shown in Table 2. This table highlights the most important attributes selected that were chosen by several weighting algorithms.

**Table 2:** The most important attributes selected by different attribute weighting algorithms.

Attributes	Sig. Test^1^	Chi Squared	Info Gain	Deviation	Gini Index	Info Gain Ratio	PCA	Correlation	Relief	Rule	Uncertainty	SVM	Mds
1. CpGn3’-UTR	***	✓	✓		✓	✓		✓	✓	✓	✓	✓	✓
2. Ala	***	✓	✓		✓	✓		✓	✓	✓		✓	✓
3 SINE 100kbUp	***	✓	✓		✓	✓		✓	✓			✓	✓
4 AvgCpG5’-UTR length	***	✓	✓		✓	✓		✓	✓	✓			✓
5 Arg	***	✓	✓		✓	✓		✓		✓			✓
6 CpGi introns	***	✓	✓		✓	✓		✓			✓		✓
7 GC introns	**	✓	✓			✓		✓	✓	✓			✓
8 N amino aicds	***		✓		✓	✓		✓		✓		✓	✓
9 CpGn gene	***				✓	✓		✓		✓		✓	✓
10CpGn introns	**	✓	✓		✓			✓			✓		✓
11 AvgCpG 100kbUp length	***	✓	✓						✓			✓	✓
12 AvgCpG3’-UTR length	**		✓		✓	✓		✓					✓
13CpGn 5’-UTR	**	✓	✓			✓		✓					✓
14 GC 10kbdwn	***	✓				✓		✓		✓			✓
15 GC 10kbup	***				✓	✓			✓	✓			✓
16 Pro	*	✓	✓		✓			✓					✓
17 SINE 10kbUp	**		✓					✓	✓			✓	✓
18 CpGi 100kbUp	**					✓		✓	✓				✓
19 GC 1kbup	***					✓		✓		✓			✓
20 CpG 100kbUp length	**		✓	✓			✓						✓

1. Significant test:

*** = very high significant p≤0.001

** = high significant p≤0.01 and

* = significant p≤0.05

**Fig 1:**
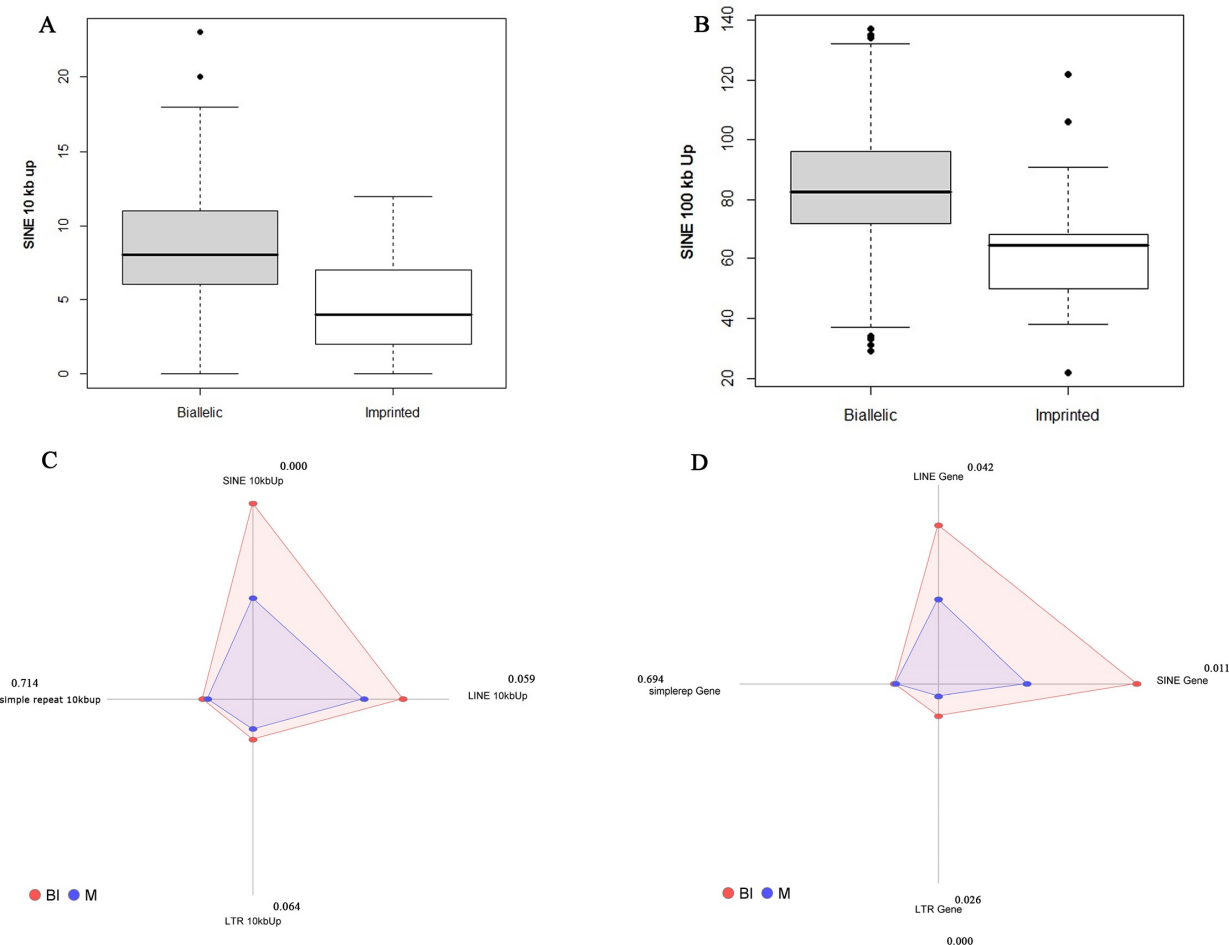
Boxplot of some important attributes of imprinted (M) and biallelic (BI) expressed genes. **(A). Boxplot of SINE 10 kb up region.** *P-*value (0.000), determined by the Mann Whitney test. As shown in the figure imprinted genes had higher SINE element in 10 kb upstream region in comparison the biallelic expressed genes. **(B). Boxplot of SINE 100 kb up region.** *P-*value (0.000), determined by the Mann Whitney test. As shown in the figure imprinted genes had higher SINE element in 100 kb upstream region in comparison the biallelic expressed genes. **(C). Web chart of repetitive elements in 10kb up region of imprinted and biallelic expressed genes.** SINE elements are significantly higher in biallelic expressed genes in comparison of imprinted genes. **(D). Web chart of repetitive elements in gene region of imprinted and biallelic expressed genes.** As shown in the chart repetitive elements of LINE, SINE and LTR are significantly higher in imprinted genes in comparison of biallelic expressed genes.

**Fig 2:**
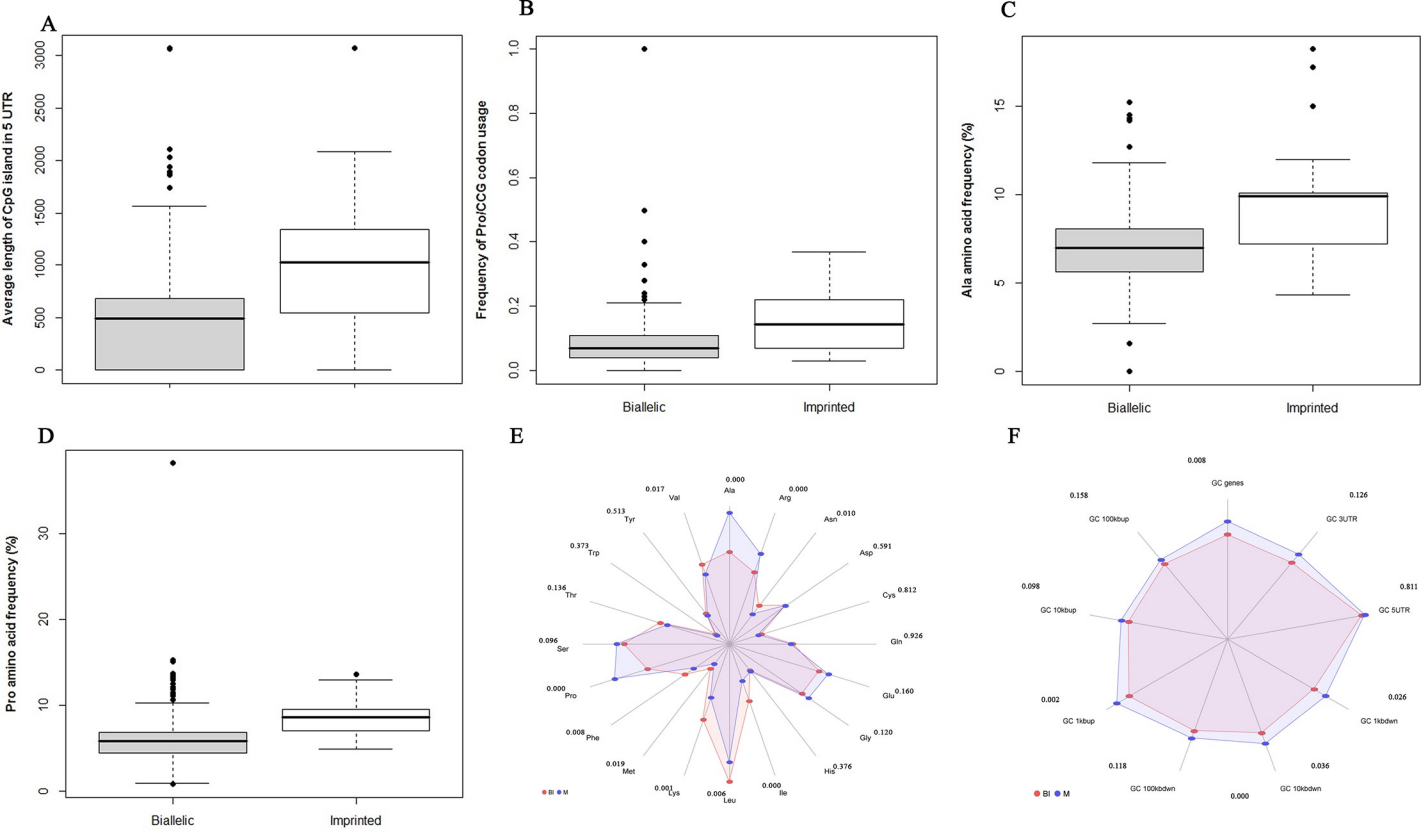
Boxplot and Web chart of some important attributes of imprinted (M) and biallelic (BI) expressed genes. **(A). Boxplot of length of CpG islands in 5 UTR,** *P-*value (0.000), determined by the Mann Whitney test. As shown in the figure imprinted genes had higher Average length CpG islands in 5’-UTR region in contrast biallelic expressed genes. **(B). Boxplot of codon usage (Pro/CCG) in imprinted (M) and biallelic (BI) expressed genes** P-value (*P<0*.*000*) determined by the Mann Whitney test. Frequency of using the Pro/CCG in the imprinted genes were higher than biallelic expressed genes. **(C). Boxplot of Ala amino acid frequency.** *P-*value (0.000), determined by the Mann Whitney test. As shown in the figure the frequency of Ala amino acid in protein sequence derived for imprinted genes was higher than the biallelic expressed genes. **(D). Boxplot of Pro amino acid frequency.** *P-*value (0.000), determined by the Mann Whitney test. As shown in the figure the frequency of Pro amino acid in protein sequence derived for imprinted genes was higher than the biallelic expressed genes. **(E). Web chart of amino acids in protein sequences of imprinted (M) and biallelic (BI) expressed genes.** P-value determined by the Mann Whitney test. The protein sequence derived attributes showed that frequency of some of the amino acids was different among imprinted and biallelic expressed genes. **(F). Web chart of GC content in several regions of imprinted (M) and biallelic (BI) expressed genes**. P-value determined by the Mann Whitney test. In the several region GC content of imprinted genes was significantly higher than biallelic expressed genes.

The frequency of SINE 10 and 100 kb up in the sequence of genes were attributes selected by these algorithms. It indicates the importance of repetitive elements in distinguishing imprinted from biallelic gene. Based on Fig 1A and 1B the imprinted genes have lower SINE in 10 and 100 kb up regions in comparison to biallelic expressed genes (*P<0*.*000*). Fig 1D shows the web chart of repetitive elements in gene region of imprinted and biallelic expressed genes. This figure demonstrates that SINE, LINE and LTR are more important than simple repeat for distinguishing biallelic from imprinted genes. Fig 1C shows the frequency of repetitive elements in 10kb up region of imprinted and biallelic expressed genes. Average length of CpG islands in 5’-UTR was also an important attribute that selected by several attribute weighting parameters (Fig 2A). Mann-Whitney U test revealed a significant difference (*P<0*.000) for average length of CpG islands in 5’-UTR between imprinted and biallelic expressed genes (S2 Table). In amino acid attributes alanin (Ala), argenine (Arg) and proline (Pro) were the most important amino acid attributes selected by algorithms. Frequency (fraction) of codon usage (Pro/CCG) in imprinted and biallelic expressed genes have been shown in Fig 2B. According to the boxplot of Ala and Pro frequencies (Fig 2C and 2D) it can be concluded that imprinted genes have higher Ala and Proin their protein sequences. The frequencies of Ala and Pro amino acids were significantly different between proteins produced by imprinted and biallelic expressed genes (*P<0*.*000*). Fig 2E, shows significant differences in the frequency of Ala, Arg, Asn, Ile, Leu, Lys, Phe, Pro and Val amino acids in protein sequence between imprinted and biallelic expressed genes.

**Fig 3:**
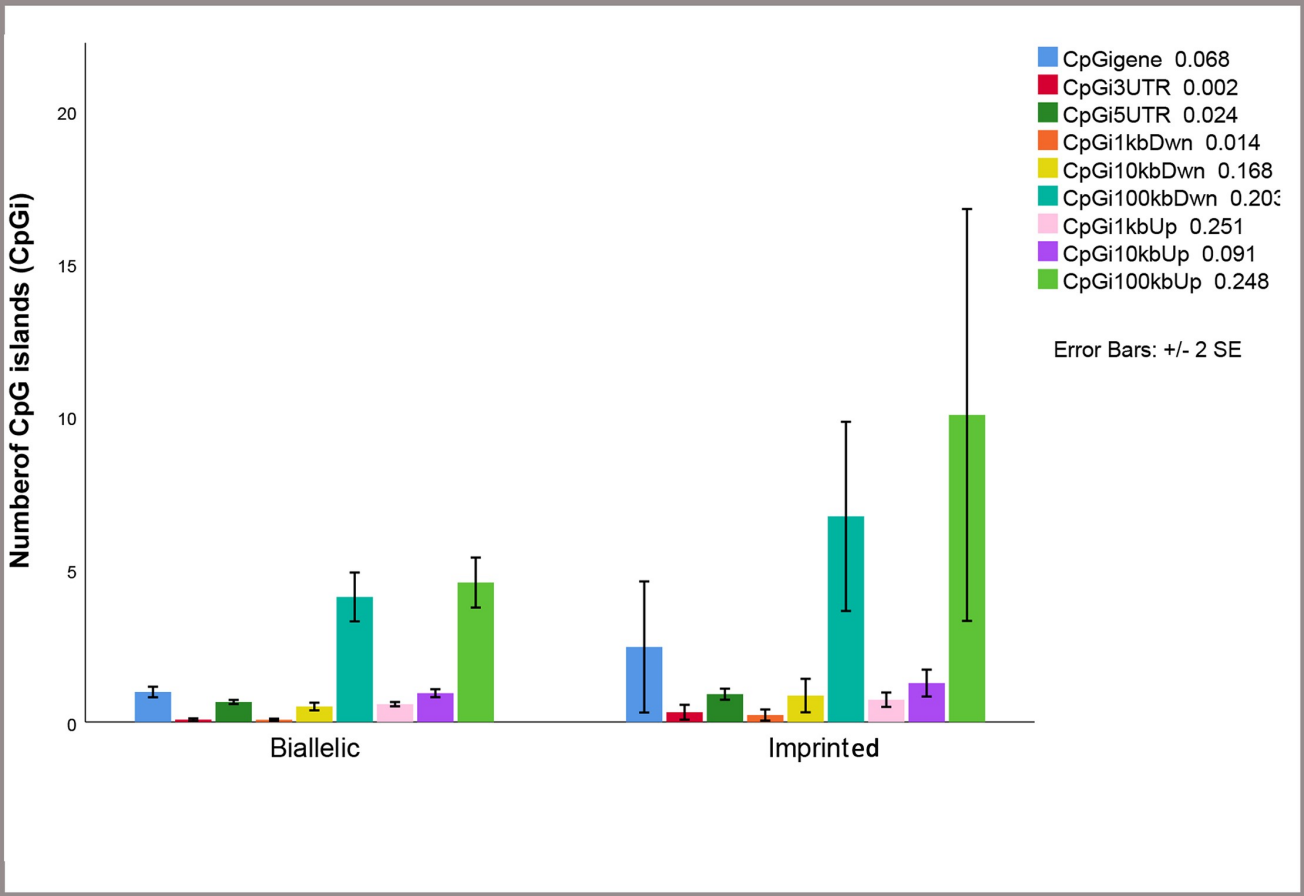
Bar plot of number of CpG (CpGi) in imprinted (M) and biallelic (BI) expressed genes. As shown in the barplot number of CpG islands was different between imprinted and biallelic expressed genes in some studied region.

**Fig 4:**
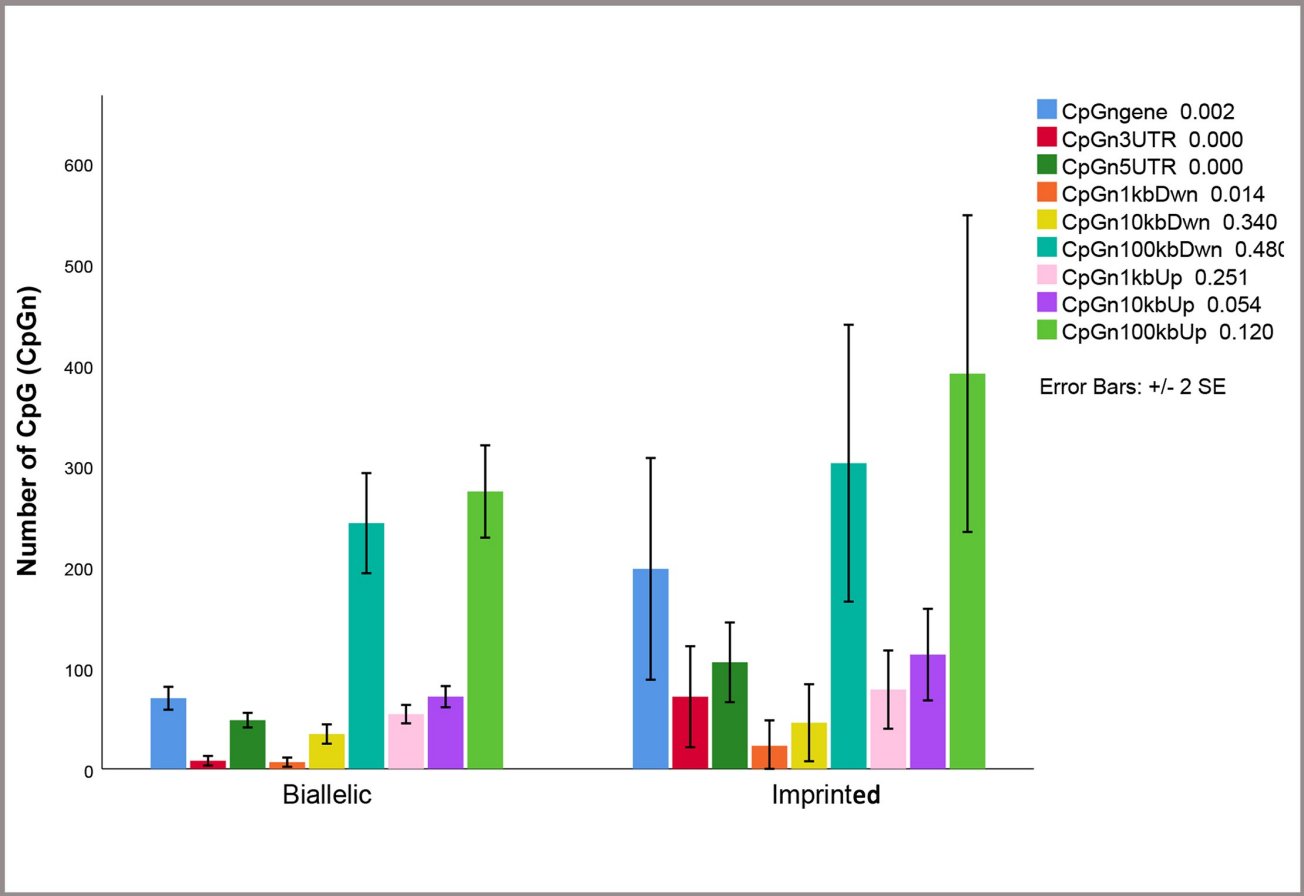
Bar plot of number of CpG (CpGn) in imprinted (M) and biallelic (BI) expressed genes. As shown in the bar plot number of CpG was different between imprinted and biallelic expressed genes in some studied region.

Figs 3 and 4 show that CpG islands and CpGn are higher in imprinted genes than biallelic genes in defined regions. Fig 4, shows barplot of CpGn in different regions. Mann-Whitney U test revealed significant differences for CpGn gene (*P<0*.002), CpGn 3’-UTR (*P<0*.000), CpGn 5’-UTR (*P<0*.000) and CpGn 1kb down (*P<0*.014) between imprinted and biallelic expressed genes.

### Supervised classification

#### Decision tree

**Fig 5:**
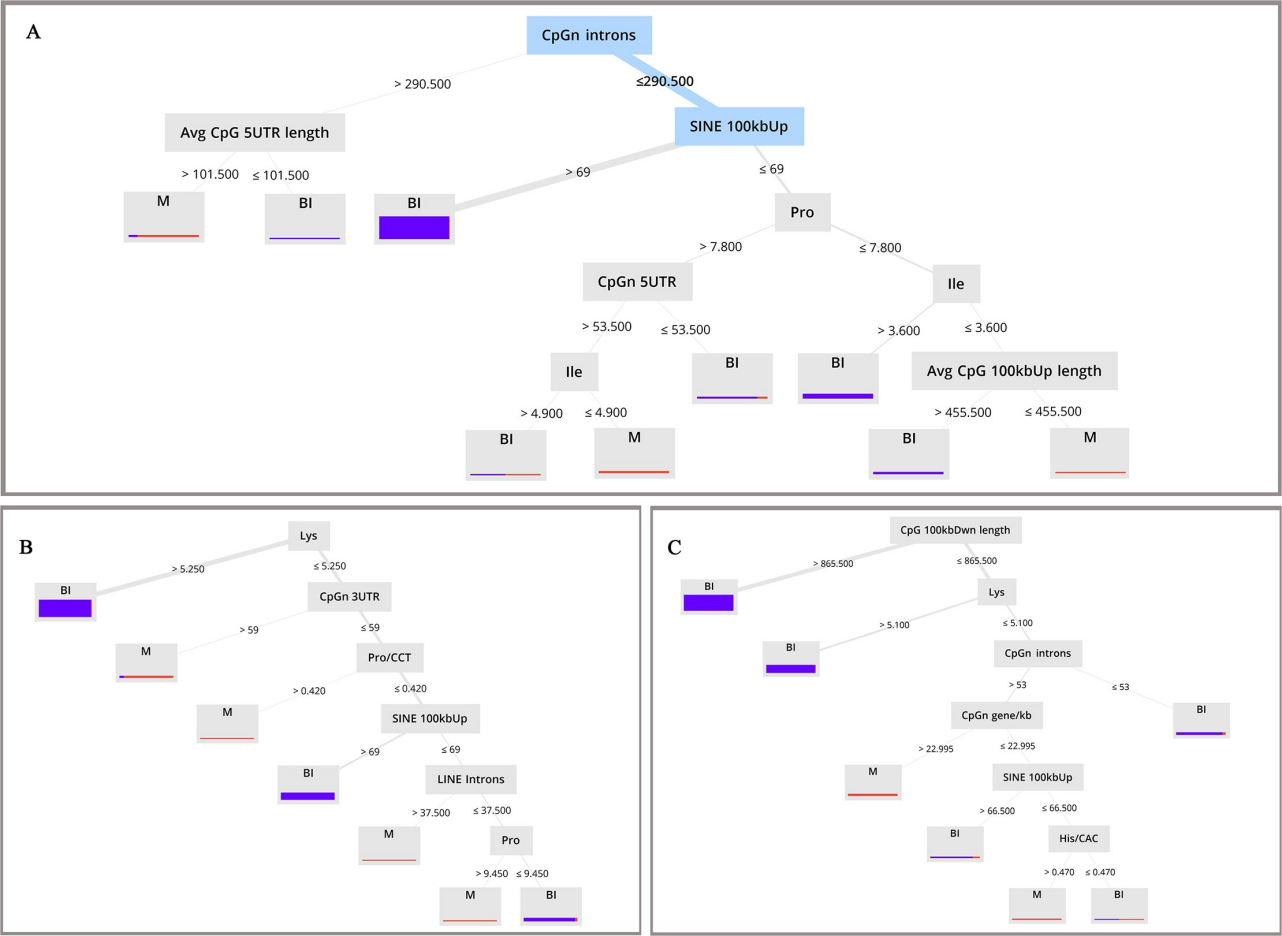
Decision Tree generated from Random Forest models with different criteria. (A). Random Forest of the Chi-Squared Statistic dataset with Information Gain criteria. (B). Random Forest of the Info Gain dataset with Gini Index criteria. (C). Random forest of the Information Gain dataset with Information Gain criteria.

Decision tree, decision stump, random tree and random forest with four criteria consisting gain ratio, information gain, Gini index and accuracy were run on the Mds and Awds. The accuracy of supervised classification algorithms on various datasets were calculated by 10-fold cross validation and was shown in Table 3 and S3 Table. The accuracy for the majority of models was greater than 90 percent. The highest accuracy was estimated by random forest with info gain criteria on the chi-squared data set (95.22) and the lowest accuracies achieved by random tree with Gini index criteria on the deviation dataset (85.65). Fig 5A, shows the random forest with info gain criteria on chi Squared dataset. This model had the highest accuracy among studied induction models (95.22), with Kappa value of 0.63, imprint recall of 50.00 and imprint precision of 100.00. Fig 5B and 5C, represents models with random forest with Gini index and info gain criteria on the Info Gain data set, respectively. The accuracies of both models were 94.35, with 40.91 and 45.45 imprint recall and 100.00 and 90.91 imprint precision, respectively. Based on algorithms of attribute weighting, the number of CpG (CpGn) in 3’-UTR region was found to be one of the main attributes (Table 2). Fig 5B, shows the application of this attribute for distinguishing imprinted and biallelic genes. Values higher than 59 indicate imprinted genes and values lower than 59 refers to biallelic expressed genes.

**Table 3:** The accuracy of four different tree induction models (each with four criteria, Accuracy, Gain Ratio, Gini Index and Information Gain) on twelve datasets computed by10-foldcross validation.

	Chi Squared	Info Gain	Deviation	Gini Index	Info Gain Ratio	PCA	Correlation	Relief	Rule	Uncertainty	SVM	Mds	Average
**Decision Tree** *Accuracy*	93.04	92.17	90.43	89.57	87.39	90.43	88.70	91.30	89.13	90.43	92.61	88.26	90.29
*Gain Ratio*	90.43	90.43	90.43	88.70	87.83	90.43	88.26	89.13	89.13	92.17	91.74	87.83	**89.71**
*Gini Index*	91.74	91.74	90.43	88.26	87.83	90.43	87.83	91.74	89.13	90.43	91.74	90.00	90.11
*Info Gain*	93.04	93.04	88.70	88.70	86.96	89.57	91.74	90.87	87.83	90.43	91.74	91.74	90.36
**Random Tree** *Accuracy*	90.00	90.43	90.43	90.00	90.43	90.43	90.00	89.57	90.43	90.00	90.43	90.43	90.22
*Gain Ratio*	92.17	90.00	90.43	91.74	89.57	90.43	91.74	90.43	90.43	90.87	92.17	89.57	90.80
*Gini Index*	91.74	90.43	**85.65**	90.87	90.87	88.70	93.04	89.57	90.87	91.30	92.61	88.70	90.36
*Info Gain*	90.87	91.30	88.70	92.17	90.00	89.57	91.74	91.30	92.61	92.17	92.17	90.00	91.05
**Decision Stump** *Accuracy*	89.13	89.57	90.43	90.00	89.57	90.43	90.43	89.13	90.00	90.00	90.87	89.57	89.93
*Gain Ratio*	89.13	89.57	90.43	90.00	89.57	90.43	90.43	89.13	90.00	90.00	90.87	89.57	89.93
*Gini Index*	90.00	90.00	90.43	90.00	90.00	90.43	90.00	90.00	90.00	91.74	90.00	90.00	90.22
*Info Gain*	89.57	89.57	90.43	89.57	89.57	90.43	89.57	89.57	89.57	91.74	89.57	89.57	89.89
**Random Forest** *Accuracy*	91.30	90.43	90.43	91.30	90.87	90.43	90.00	90.43	91.30	88.70	90.87	90.43	90.54
*Gain Ratio*	91.30	93.04	90.43	89.57	92.17	90.43	90.00	90.87	90.87	88.26	92.61	90.43	90.83
*Gini Index*	93.91	94.35	90.43	92.17	92.61	89.13	90.87	91.74	93.04	90.00	93.04	91.74	91.92
*Info Gain*	**95.22**	94.35	88.70	92.61	92.17	88.70	93.04	92.61	93.04	90.43	94.35	93.04	**92.36**
**Average**	91.41	91.28	**89.81**	90.33	89.84	90.03	90.46	90.46	90.46	90.54	**91.71**	90.06	

This table presents the accuracy percentage of Tree Induction models (*Decision Tree*, *Random Tree*, *Decision Stump and Random Forest*) with four different criteria (*Accuracy*, *Gain Ratio*, *Gini Index and Information Gain*). The lowest and the highest accuracies are bold.

#### Neural network and Bayesian models

Table 4 presents the performance of different neural network algorithms and Bayesian models using provided data sets. These models had an average accuracy of 86 percent. The highest predicted accuracy achieved by W-BayesNet on the SVM dataset (96.52%) and the lowest accuracies achieved by perceptron on the PCA data set (29.13%). W-Bayes Net on SVM were performed with Kappa value of 0.763, imprint recall of 72.73, and imprint precision of 88.89 (S4 Table).

**Table 4:** The accuracy of neural network and Bayesian models on twelve datasets computed by 10-foldcross validation.

	Chi Squared	Info Gain	Deviation	Gini Index	Info Gain Ratio	PCA	Correlation	Relief	Rule	Uncertainty	SVM	Mds	Average
**Neural Network** *Deep Learning*	90.82	93.51	59.57	89.81	88.59	63.21	91.06	88.77	88.34	87.15	94.60	91.67	85.59
*AutoMPL*	90.43	94.35	90.43	90.87	90.00	90.43	91.74	90.00	91.30	90.43	95.22	91.74	91.41
*Neural net*	90.00	93.04	90.43	90.87	90.00	90.43	90.00	88.26	89.13	88.70	94.35	91.30	90.54
*perceptron*	81.30	93.91	44.78	83.48	87.83	**29.13**	90.00	88.26	69.57	42.61	90.43	92.61	**74.49**
**Bayesian Models** *Naive Bayes*	89.13	91.74	61.30	88.26	86.96	63.48	88.26	83.04	86.52	90.00	93.48	87.83	84.17
*BayesKernel*	91.74	96.09	60.00	90.87	90.00	59.13	91.74	88.26	90.87	91.74	94.78	90.43	86.30
*W-BayesNet*	93.48	95.22	90.43	92.61	93.91	90.43	94.35	95.22	92.61	90.00	**96.52**	95.22	**93.33**
**Average**	89.56	93.98	70.99	89.54	89.61	**69.46**	91.02	88.83	86.91	82.95	**94.20**	91.54	

This table presents the accuracy percentage of Neural Network models (*Deep Learning*, *AutoMPL*, *Neural net and perceptron*) and Bayesian Models (*Naive Bayes*, *Bayes Kernel and W-BayesNet*). The lowest and highest accuracies are bolded.

### Unsupervised clustering

Unsupervised clustering algorithms consisting K-means and K-Medoids were implemented on the Mds and Awds. Each kind of clustering was not able to completely distinguish between mono (imprinted) and biallelic expressed genes. The accuracy of unsupervised clustering algorithms on various datasets obtained by 10-fold cross validation and was shown in Table 5.

**Table 5:** Clustering of 12 datasets (Mds and Awds) into MONO and BI classes by different unsupervised clustering algorithms (K-Means and K-Medoids).

Dataset	Type of Expression	K- Means		K-Medoids	
		predicted	True predicted	predicted	True predicted
**Chi Squared**	Mono: 22	38	9	5	2
	Bi: 208	192	179	225	205
**Deviation**	Mono: 22	1	0	10	0
	Bi: 208	229	207	220	198
**Gini Index**	Mono: 22	36	12	4	2
	Bi: 208	194	184	226	206
**Information Gain**	Mono: 22	1	0	5	2
	Bi: 208	229	207	225	205
**Information Gain Ratio**	Mono: 22	24	4	6	2
	Bi: 208	206	188	224	204
**PCA**	Mono: 22	1	0	74	0
	Bi: 208	229	207	156	134
**Correlation**	Mono: 22	32	6	6	2
	Bi: 208	198	182	224	204
**Relief**	Mono: 22	35	9	6	3
	Bi: 208	195	182	224	205
**Rule**	Mono: 22	27	5	25	7
	Bi: 208	203	186	205	190
**SVM**	Mono: 22	26	0	47	0
	Bi: 208	204	182	183	161
**Uncertainty**	Mono: 22	8	5	9	5
	Bi: 208	222	205	221	204
**Mds**	Mono: 22	1	0	10	0
	Bi: 208	229	207	220	198

#### Test accuracy of classifiers

To test the accuracy of the classifiers on a new set of genes, the imprinted bovine genes identified by Chen Z et al (2016) were used. 50 classifiers with accuracy more than 92 percent were tested on 35 genes that identified as imprinted by Chen Z et al (2016) [64]. Number of classifiers successfully detected gene as imprinting shown in Table 6 and S5 Table. We found that except for 5 genes, our models were able to classify the remaining genes as imprinted.

**Table 6:** Number of classifiers successfully detected the gene as imprinted.

Gene ID	Gene symbol	Number of classifiers successfully detected the gene as imprinting
ENSBTAG00000023338	PEG3	48
ENSBTAG00000031184	CDKN1C	47
ENSBTAG00000013066	IGF2	47
ENSBTAG00000002402	IGF2R	39
ENSBTAG00000038093	PEG10	25
ENSBTAG00000010128	NAP1L5	21
ENSBTAG00000031194	PHLDA2	20
ENSBTAG00000046585	RTL1	19
ENSBTAG00000017716	BEGAIN	18
-	H19	16
ENSBTAG00000021282	SGCE	13
-	MEG8	10
ENSBTAG00000026523	PLAGL1	8
-	MEG9	8
ENSBTAG00000019600	OOEP	6
ENSBTAG00000017475	GNAS	3
ENSBTAG00000001392	RDH16	3
ENSBTAG00000008251	SNRPN	3
ENSBTAG00000009725	AOX1	2
ENSBTAG00000019616	APCS	2
ENSBTAG00000037899	DLK1	2
ENSBTAG00000048005	MGC157368	2
-	MEG3	2
ENSBTAG00000007975	ALDH8A1	1
ENSBTAG00000004840	C1S	1
ENSBTAG00000012182	DIRAS3	1
ENSBTAG00000034645	PON3	1
ENSBTAG00000024426	PPP1R9A	1
ENSBTAG00000005386	SLC2A2	1
ENSBTAG00000038326	LOC508098	1
ENSBTAG00000036127	AS3MT	0
ENSBTAG00000008612	C1R	0
ENSBTAG00000006136	CDA	0
ENSBTAG00000016165	KRT7	0
ENSBTAG00000015074	PTGDS	0

## Discussion

SVM dataset with the average accuracy of 91.71% in induction models and 94.20% in Neural Network and Bayesian models had the highest accuracy among evaluated datasets. Therefore, this pattern could be better than others in distinguishing imprinted and biallelic expressed genes. This dataset comprised as the length of CpG in 100 kb down, average length of CpG in 100 kb down, SINE 100 kb UP, CpGi 3’-UTR, Ala, average length of CpG in gene region, SINE 10 kb up, CpGn 3’-UTR, average length of CpG 10 kb down and Pro/CCT.

The performance of gene expression is influenced by the composition of the nucleotide in the coding region like GC content and codon usage. Although, these two important attributes are strongly connected to gene expression, the molecular functions behind these attributes are not completely obvious[65]. The GC content of imprinted genes in several regions was higher than biallelic expressed genes (Fig 2F). These observations are in agreement with the results presented by Khatib et al. (2007). However, in contrast to these results, it was reported by Walter, et al. (2006) that the GC content of genes with imprinted expression is like a subset of genes in the mouse genome which are randomly selected. In addition, Hutter et al. (2006) compared human and mouse genomes and reported no statistically significant differences between control and imprinted genes in these species. Such contradiction might indicate a different evolutionary pathway for cattle genome compared to those of mouse and human.

Furthermore, previous studies suggested a positive correlation between codon usage bias and the level of gene expression [32, 65, 66]. It could be an important element for the stability of mRNA and identification of translational efficiency [65]. Also in some species, it has been shown that there is a correlation between preference of codon usage and the abundance of the respective tRNA [67–71] which at last can affect translational efficiency of the gene products [70]. Fig 1D shows the boxplot of codon usage (Pro/CCG) in imprinted (M) and biallelic (BI) expressed genes.

Differences in the occurrence of repeated sequences between biallelic expressed genes and imprinted genes have been reported in several studies. Usually, high CpG islands, transcription factor binding sites (TFBS) and repetitive elements are the sequence characteristics of imprinted genes[72, 73]. For analyzing known and hypothetical imprinted genes, these attributes are normally used [3, 19]. Fewer and smaller introns can be seen in imprinted genes compared to non-imprinted genes. They have also some degrees of repetitive sequences and contain an unusually high number of retrotransposable elements [74]. In present study intron counts in imprinted and biallelic expressed genes were not significantly different (*P<0*.184). However, imprinted genes showed significantly lower frequencies of SINE in 10 and 100 kb up compared to biallelic expressed genes in bovine. Depletion of SINEs is adapted with reasonably increased frequency of LINEs in imprinted genes. Interestingly, Cowley et al. (2011) found no significant differences in LINE or SINE in the mono and biallelic expressed genes neither in mouse nor human. In the study of Greally (2002) several sequence attributes of human imprinted genes were compared to non-imprinted genes. He demonstrated that fewer SINE transposons-derived sequences can be seen in imprinted loci than biallelic loci and the number of SINEs is directly correlated with the imprinting status.

Additionally, the frequency of SINE in the flanking regions of biallelic and imprinted genes is low (Allen et al., 2003). Cowley et al. (2011) suggested that LINEs and SINEs are genomic attributes which are not directly correlated with imprinting status of genes. Khatib et al. (2007) found higher GC content, CpG islands, and tandem repeats in imprinted genes than non-imprinted genes in bovine. Additionally, the frequency of SINEs in imprinted genes of bovine was lower than biallelic expressed genes. These findings are in agreement with findings in human and mouse. In accordance with Khatib et al. (2007), the number of LINEs and LTRs were found to be significantly lower in imprinted genes compared to biallelic genes in present study (Fig 3B) which are in contrast to the findings in human and mouse. Allen et al, (2003) used cluster analysis to examine heterogeneity in monoallelic genes in human and mouse with respect to sequence attributes. They found biallelic expressed genes display lower number of LINE-1 sequence, while the imprinted genes were flanked by lower amounts of SINE sequence [31]. Weidman et al. (2004) reported that the imprinting of IGF2 is strongly associated with the lack of SINEs. Similarly, Walter et al. (2006) showed that the frequency of SINEs in imprinted genes (7.32%) is lower than non-imprinted genes (13.9%). They also reported higher GC content and higher number of LINEs sequences in the imprinted genes compared to non- imprinted genes.

The pattern of distribution of repetitive elements, in combination with other sequence attributes, has been used for prediction of putative imprinted genes in the mouse [17] and human genomes [18]. Some studies showed that in human genome, SINE elements are enriched in GC- and gene-rich regions, whereas LINE elements harbor lower GC and are mostly found in gene-poor regions [75, 76]. Tandem repeats have been implicated in the regulation of mouse imprinted genes including *Rasgrf1*, *Xist*, and *Tsix* [77–79]. Although the mechanism of imprinted methylation through tandem repeats is unknown, hypotheses such as siRNA mediated regulation, secondary structure formation, and involvement of germline-specific repeat binding factors could be take into account. Tandem repeats might continuously produce siRNA through the use of RNA-dependent RNA polymerase (RdRP) and cause multiple rounds of RNA interference (RNAi) [80]. Such events have been detected in the regulation of epigenetic phenomena in fission yeast and plants and may operate in mice as well [81–84]. Therefore, these attributes may affect the establishment or maintenance of DNA methylation at imprinted loci during development, especially during germline development.

## Conclusions

According to our results, attributes related to GC content and CpG in upstream and downstream regions of genes, SINE in 10 and 100kbUp and frequency of some amino acids including Ala, Arg, Pro were the most important attributes for distinguish imprinted and biallelic expressed genes. The sequence characteristics presented in the current study predict the imprinting status of genes in bovine with high accuracy. This method could be applied to expand the number of imprinted genes in genome of other species. With more imprinted genes in hand, it would be possible to deepen our understandings regarding the genetic and epigenetic regulatory mechanism involved in the monoallelic expression of imprinted genes. Besides, assessment of the method in other genomes might be useful to find an evolutionary relationship among species and would be beneficial to find monoallelically expressed genes elsewhere. Also, the next step would be the application of these patterns in the identification of novel sets of imprinted genes.

## Supporting information

S1 Table Initial dataset (raw dataset). (XLSX)

S2 Table Nonparametric tests. (XLSX)

S3 Table The kappa value, accuracies, biallelic and imprint recalls and precisions of induction models computed on 10-fold CV. (DOCX)

S4 Table The kappa value, accuracies, biallelic and imprint recalls and precisions of Neural Network and Bayesian models computed on 10-fold CV. (DOCX)

S5 Table Classifiers successfully detected the gene as imprinted. (XLSX)

## References

[1] Tian XC. Bovine Epigenetics and Epigenomics. Bovine Genomics. 2012:144.

[2] Tian F, Tang Z, Song G, Pan Y, He B, Bao Q, et al Loss of imprinting of IGF2 correlates with hypomethylation of the H19 differentially methylated region in the tumor tissue of colorectal cancer patients. Molecular medicine reports. 2012;5(6):1536–40. 10.3892/mmr.2012.833 22427002

[3] Khatib H, Zaitoun I, Kim E-S. Comparative analysis of sequence characteristics of imprinted genes in human, mouse, and cattle. Mammalian Genome. 2007;18(6–7):538–47. 10.1007/s00335-007-9039-z 17653590 PMC2000230

[4] Feinberg A. DNA methylation, genomic imprinting and cancer. Current topics in microbiology and immunology. 2000;249:87–99. 10802940 10.1007/978-3-642-59696-4_6

[5] Surani MA. Reprogramming of genome function through epigenetic inheritance. Nature. 2001;414(6859):122–9. 10.1038/35102186 11689958

[6] Nicholls RD, Knepper JL. Genome organization, function, and imprinting in Prader-Willi and Angelman syndromes. Annual review of genomics and human genetics. 2001;2(1):153–75. 10.1146/annurev.genom.2.1.153 11701647

[7] Mannens M, Hoovers J, Redeker E, Verjaal M, Feinberg A, Little P, et al Parental imprinting of human chromosome region 11p15. 3-pter involved in the Beckwith-Wiedemann syndrome and various human neoplasia. European Journal of Human Genetics. 1994;2:3–23. 10.1159/000472337 7913866

[8] Ogawa O, Eccles MR, Szeto J, McNoe LA, Yun K, Maw MA, et al Relaxation of insulin-like growth factor II gene imprinting implicated in Wilms' tumour. Nature. 1993;362(6422):749–51. 10.1038/362749a0 8097018

[9] Rainier S, Johnson LA, Dobry CJ, Ping AJ, Grundy PE, Feinberg AP. Relaxation of imprinted genes in human cancer. 1993. 10.1038/362747a0 8385745

[10] Chen Z, Hagen DE, Elsik CG, Ji T, Morris CJ, Moon LE, et al Characterization of global loss of imprinting in fetal overgrowth syndrome induced by assisted reproduction. Proceedings of the National Academy of Sciences. 2015;112(15):4618–23. 10.1073/pnas.1422088112 PMC4403167 25825726

[11] Plasschaert RN, Bartolomei MS. Genomic imprinting in development, growth, behavior and stem cells. Development. 2014;141(9):1805–13. 10.1242/dev.101428 24757003 PMC3994769

[12] Barlow DP. Genomic imprinting: a mammalian epigenetic discovery model. Annual review of genetics. 2011;45:379–403. 10.1146/annurev-genet-110410-132459 21942369

[13] Koerner MV, Barlow DP. Genomic imprinting—an epigenetic gene-regulatory model. Current opinion in genetics & development. 2010;20(2):164–70. 20153958 10.1016/j.gde.2010.01.009 PMC2860637

[14] Cowley M, De Burca A, McCole RB, Chahal M, Saadat G, Oakey RJ, et al Short interspersed element (SINE) depletion and long interspersed element (LINE) abundance are not features universally required for imprinting. PloS one. 2011;6(4):e18953 10.1371/journal.pone.0018953 21533089 PMC3080381

[15] Moore GE, Ishida M, Demetriou C, Al-Olabi L, Leon LJ, Thomas AC, et al The role and interaction of imprinted genes in human fetal growth. Philosophical Transactions of the Royal Society B: Biological Sciences. 2015;370(1663):20140074. 10.1098/rstb.2014.0074 PMC4305174 25602077

[16] Piedrahita JA. The role of imprinted genes in fetal growth abnormalities. Birth Defects Research Part A: Clinical and Molecular Teratology. 2011;91(8):682–92. 10.1002/bdra.20795 21648055 PMC3189628

[17] Luedi PP, Hartemink AJ, Jirtle RL. Genome-wide prediction of imprinted murine genes. Genome research. 2005;15(6):875–84. 10.1101/gr.3303505 15930497 PMC1142478

[18] Luedi PP, Dietrich FS, Weidman JR, Bosko JM, Jirtle RL, Hartemink AJ. Computational and experimental identification of novel human imprinted genes. Genome research. 2007;17(12):1723–30. 10.1101/gr.6584707 18055845 PMC2099581

[19] Nikaido I, Saito C, Mizuno Y, Meguro M, Bono H, Kadomura M, et al Discovery of imprinted transcripts in the mouse transcriptome using large-scale expression profiling. Genome Research. 2003;13(6b):1402–9. 10.1101/gr.1055303 12819139 PMC403673

[20] Imumorin IG, Peters SO, De Donato M. Genomic imprinting and imprinted gene clusters in the bovine genome. Livestock Epigenetics. 2012:89–111.

[21] Jirtle RL. Geneimprint. Available online at: http://www.geneimprint.org. 2012.

[22] Wei Y, Su J, Liu H, Lv J, Wang F, Yan H, et al MetaImprint: an information repository of mammalian imprinted genes. Development. 2014;141(12):2516–23. 10.1242/dev.105320 24850854

[23] Wang X, Clark A. Using next-generation RNA sequencing to identify imprinted genes. Heredity. 2014;113(2):156–66. 10.1038/hdy.2014.18 24619182 PMC4105452

[24] Uyar A, Seli E. The impact of assisted reproductive technologies on genomic imprinting and imprinting disorders. Current opinion in obstetrics & gynecology. 2014;26(3):210. 24752003 10.1097/GCO.0000000000000071 PMC4123998

[25] Yang X, Shao X, Gao L, Zhang S. Comparative DNA methylation analysis to decipher common and cell type-specific patterns among multiple cell types. Briefings in functional genomics. 2016;15(6):399–407. 10.1093/bfgp/elw013 27107288

[26] Gardiner-Garden M, Frommer M. CpG islands in vertebrate genomes. Journal of molecular biology. 1987;196(2):261–82. 3656447 10.1016/0022-2836(87)90689-9

[27] Toyota M, Ahuja N, Ohe-Toyota M, Herman JG, Baylin SB, Issa J-PJ. CpG island methylator phenotype in colorectal cancer. Proceedings of the National Academy of Sciences. 1999;96(15):8681–6. 10.1073/pnas.96.15.8681 PMC17576 10411935

[28] Zeng J, Yi SV. DNA methylation and genome evolution in honeybee: gene length, expression, functional enrichment covary with the evolutionary signature of DNA methylation. Genome biology and evolution. 2010;2:770–80. 10.1093/gbe/evq060 20924039 PMC2975444

[29] Reed MR, Riggs AD, Mann JR. Deletion of a direct repeat element has no effect on Igf2 and H19 imprinting. Mammalian genome. 2001;12(11):873–6. 11845292 10.1007/s00335-001-2027-9

[30] Lewis A, Mitsuya K, Constancia M, Reik W. Tandem repeat hypothesis in imprinting: deletion of a conserved direct repeat element upstream of H19 has no effect on imprinting in the Igf2-H19 region. Molecular and cellular biology. 2004;24(13):5650–6. 10.1128/MCB.24.13.5650-5656.2004 15199123 PMC480907

[31] Allen E, Horvath S, Tong F, Kraft P, Spiteri E, Riggs AD, et al High concentrations of long interspersed nuclear element sequence distinguish monoallelically expressed genes. Proceedings of the National Academy of Sciences. 2003;100(17):9940–5. 10.1073/pnas.1737401100 PMC187893 12909712

[32] Hiraoka Y, Kawamata K, Haraguchi T, Chikashige Y. Codon usage bias is correlated with gene expression levels in the fission yeast Schizosaccharomyces pombe. Genes to Cells. 2009;14(4):499–509. 10.1111/j.1365-2443.2009.01284.x 19335619

[33] Zhou Z, Dang Y, Zhou M, Li L, Yu C-h, Fu J, et al Codon usage is an important determinant of gene expression levels largely through its effects on transcription. Proceedings of the National Academy of Sciences. 2016;113(41):E6117–E25. 10.1073/pnas.1606724113 PMC5068308 27671647

[34] Hastie T, Tibshirani R, Friedman J. The elements of statistical learning: data mining, inference, and prediction 2 edition Springer New York 2009.

[35] Murphy KP. Machine learning: a probabilistic perspective: MIT press; 2012.

[36] Michalski RS, Carbonell JG, Mitchell TM. Machine learning: An artificial intelligence approach: Springer Science & Business Media; 2013.

[37] Angermueller C, Lee H, Reik W, Stegle O. Accurate prediction of single-cell DNA methylation states using deep learning. bioRxiv. 2017:055715. 10.1186/s13059-017-1189-z PMC5387360 28395661

[38] Zycinski G, Barla A, Verri A, editors. SVS: Data and knowledge integration in computational biology. Engineering in Medicine and Biology Society, EMBC, 2011 Annual International Conference of the IEEE; 2011: IEEE. 10.1109/IEMBS.2011.6091598 22255821

[39] Zeng X, Martinez TR, editors. Feature weighting using neural networks. Neural Networks, 2004 Proceedings 2004 IEEE International Joint Conference on; 2004: IEEE.

[40] Thai K-M, Ecker GF. Similarity-based SIBAR descriptors for classification of chemically diverse hERG blockers. Molecular diversity. 2009;13(3):321–36. 10.1007/s11030-009-9117-0 19219559

[41] Ebrahimi M, Lakizadeh A, Agha-Golzadeh P, Ebrahimie E, Ebrahimi M. Prediction of thermostability from amino acid attributes by combination of clustering with attribute weighting: a new vista in engineering enzymes. PloS one. 2011;6(8):e23146 10.1371/journal.pone.0023146 21853079 PMC3154288

[42] Beiki AH, Saboor S, Ebrahimi M. A new avenue for classification and prediction of olive cultivars using supervised and unsupervised algorithms. PloS one. 2012;7(9):e44164 10.1371/journal.pone.0044164 22957050 PMC3434224

[43] Hosseinzadeh F, Ebrahimi M, Goliaei B, Shamabadi N. Classification of lung cancer tumors based on structural and physicochemical properties of proteins by bioinformatics models. PLoS One. 2012;7(7):e40017 10.1371/journal.pone.0040017 22829872 PMC3400626

[44] Bai L, Liang J, Dang C, Cao F. A novel attribute weighting algorithm for clustering high-dimensional categorical data. Pattern Recognition. 2011;44(12):2843–61.

[45] Abeel T, Saeys Y, Rouzé P, Van de Peer Y. ProSOM: core promoter prediction based on unsupervised clustering of DNA physical profiles. Bioinformatics. 2008;24(13):i24–i31. 10.1093/bioinformatics/btn172 18586720 PMC2718650

[46] Velmurugan T, Santhanam T. Computational complexity between K-means and K-medoids clustering algorithms for normal and uniform distributions of data points. Journal of computer science. 2010;6(3):363.

[47] MacQueen J, editor Some methods for classification and analysis of multivariate observations. Proceedings of the fifth Berkeley symposium on mathematical statistics and probability; 1967: Oakland, CA, USA.

[48] Plath K, Mlynarczyk-Evans S, Nusinow DA, Panning B. Xist RNA and the mechanism of X chromosome inactivation. Annual review of genetics. 2002;36(1):233–78. 10.1146/annurev.genet.36.042902.092433 12429693

[49] Takagi N, Sasaki M. Preferential inactivation of the paternally derived X chromosome in the extraembryonic membranes of the mouse. Nature. 1975;256(5519):640–2. 1152998 10.1038/256640a0

[50] Spitzer M, Wildenhain J, Rappsilber J, Tyers M. BoxPlotR: a web tool for generation of box plots. Nature methods. 2014;11(2):121–2. 10.1038/nmeth.2811 24481215 PMC3930876

[51] RapidMiner Technical Support Available from: http://docs.rapidminer.com/.

[52] Rokach L, Maimon O. Classification trees. Data mining and knowledge discovery handbook: Springer; 2009 p. 149–74.

[53] Eickholt J, Cheng J. DNdisorder: predicting protein disorder using boosting and deep networks. BMC bioinformatics. 2013;14(1):88. 23497251 10.1186/1471-2105-14-88 PMC3599628

[54] Dahl GE, Jaitly N, Salakhutdinov R. Multi-task neural networks for QSAR predictions. arXiv preprint arXiv:14061231 2014.

[55] Leung MK, Xiong HY, Lee LJ, Frey BJ. Deep learning of the tissue-regulated splicing code. Bioinformatics. 2014;30(12):i121–i9. 10.1093/bioinformatics/btu277 24931975 PMC4058935

[56] Sønderby SK, Winther O. Protein secondary structure prediction with long short term memory networks. arXiv preprint arXiv:14127828 2014.

[57] Alipanahi B, Delong A, Weirauch MT, Frey BJ. Predicting the sequence specificities of DNA-and RNA-binding proteins by deep learning. Nature biotechnology. 2015;33(8):831–8. 10.1038/nbt.3300 26213851

[58] Wang K, Cao K, Hannenhalli S, editors. Chromatin and genomic determinants of alternative splicing. Proceedings of the 6th ACM Conference on Bioinformatics, Computational Biology and Health Informatics; 2015: ACM. 10.1145/2808719.2808755 PMC5558438 28825057

[59] Zhou J, Troyanskaya OG. Predicting effects of noncoding variants with deep learning–based sequence model. Nature methods. 2015;12(10):931 10.1038/nmeth.3547 26301843 PMC4768299

[60] Kelley DR, Snoek J, Rinn JL. Basset: learning the regulatory code of the accessible genome with deep convolutional neural networks. Genome research. 2016;26(7):990–9. 10.1101/gr.200535.115 27197224 PMC4937568

[61] Domingos P, Pazzani M. On the optimality of the simple Bayesian classifier under zero-one loss. Machine learning. 1997;29(2):103–30.

[62] McCallum A, Nigam K, editors. A comparison of event models for naive bayes text classification. AAAI-98 workshop on learning for text categorization; 1998: Madison, WI.

[63] Tan S, Zhang J. An empirical study of sentiment analysis for chinese documents. Expert Systems with applications. 2008;34(4):2622–9.

[64] Chen Z, Hagen DE, Wang J, Elsik CG, Ji T, Siqueira LG, et al Global assessment of imprinted gene expression in the bovine conceptus by next generation sequencing. Epigenetics. 2016;11(7):501–16. 10.1080/15592294.2016.1184805 27245094 PMC4939914

[65] Barahimipour R, Strenkert D, Neupert J, Schroda M, Merchant SS, Bock R. Dissecting the contributions of GC content and codon usage to gene expression in the model alga Chlamydomonas reinhardtii. The Plant Journal. 2015;84(4):704–17. 10.1111/tpj.13033 26402748 PMC4715772

[66] Wang L, Roossinck MJ. Comparative analysis of expressed sequences reveals a conserved pattern of optimal codon usage in plants. Plant molecular biology. 2006;61(4):699–710. 16897485 10.1007/s11103-006-0041-8

[67] Ikemura T. Codon usage and tRNA content in unicellular and multicellular organisms. Molecular biology and evolution. 1985;2(1):13–34. 10.1093/oxfordjournals.molbev.a040335 3916708

[68] Andersson S, Kurland C. Codon preferences in free-living microorganisms. Microbiological reviews. 1990;54(2):198–210. 2194095 10.1128/mr.54.2.198-210.1990 PMC372768

[69] Sharp PM, Stenico M, Peden JF, Lloyd AT. Codon usage: mutational bias, translational selection, or both?: Portland Press Limited; 1993. 10.1042/bst0210835 8132077

[70] Akashi H. Translational selection and yeast proteome evolution. Genetics. 2003;164(4):1291–303. 12930740 10.1093/genetics/164.4.1291 PMC1462678

[71] Kanaya S, Yamada Y, Kinouchi M, Kudo Y, Ikemura T. Codon usage and tRNA genes in eukaryotes: correlation of codon usage diversity with translation efficiency and with CG-dinucleotide usage as assessed by multivariate analysis. Journal of molecular evolution. 2001;53(4):290–8. 11675589 10.1007/s002390010219

[72] Paulsen M, El-Maarri O, Engemann S, Strödicke M, Franck O, Davies K, et al Sequence conservation and variability of imprinting in the Beckwith–Wiedemann syndrome gene cluster in human and mouse. Human molecular genetics. 2000;9(12):1829–41. 10.1093/hmg/9.12.1829 10915772

[73] Neumann B, Kubicka P, Barlow DP. Characteristics of imprinted genes. Nature genetics. 1995;9(1):12–3. 10.1038/ng0195-12 7704015

[74] Armstrong L. Epigenetics: Garland science; 2013.

[75] Lander ES, Linton LM, Birren B, Nusbaum C, Zody MC, Baldwin J, et al Initial sequencing and analysis of the human genome. 2001 10.1038/35057062 11237011

[76] Smit AF. Interspersed repeats and other mementos of transposable elements in mammalian genomes. Current opinion in genetics & development. 1999;9(6):657–63. 10607616 10.1016/s0959-437x(99)00031-3

[77] Yoon HR, Park YS, Kim YK. Rapid prenatal detection of Down and Edwards syndromes by fluorescent polymerase chain reaction with short tandem repeat markers. Yonsei Medical Journal. 2002;43(5):557–66. 10.3349/ymj.2002.43.5.557 12402367

[78] Hoki Y, Kimura N, Kanbayashi M, Amakawa Y, Ohhata T, Sasaki H, et al A proximal conserved repeat in the Xist gene is essential as a genomic element for X-inactivation in mouse. Development. 2009;136(1):139–46. 10.1242/dev.026427 19036803

[79] Cohen DE, Davidow LS, Erwin JA, Xu N, Warshawsky D, Lee JT. The DXPas34 repeat regulates random and imprinted X inactivation. Developmental cell. 2007;12(1):57–71. 10.1016/j.devcel.2006.11.014 17199041

[80] Martienssen RA. Maintenance of heterochromatin by RNA interference of tandem repeats. Nature genetics. 2003;35(3):213–5. 10.1038/ng1252 14593407

[81] Alleman M, Sidorenko L, McGinnis K, Seshadri V, Dorweiler JE, White J, et al An RNA-dependent RNA polymerase is required for paramutation in maize. Nature. 2006;442(7100):295–8. 10.1038/nature04884 16855589

[82] Chan SW-L, Zilberman D, Xie Z, Johansen LK, Carrington JC, Jacobsen SE. RNA silencing genes control de novo DNA methylation. Science. 2004;303(5662):1336–. 10.1126/science.1095989 14988555

[83] Rassoulzadegan M, Grandjean V, Gounon P, Vincent S, Gillot I, Cuzin F. RNA-mediated non-mendelian inheritance of an epigenetic change in the mouse. Nature. 2006;441(7092):469–74. 10.1038/nature04674 16724059

[84] Verdel A, Jia S, Gerber S, Sugiyama T, Gygi S, Grewal SI, et al RNAi-mediated targeting of heterochromatin by the RITS complex. Science. 2004;303(5658):672–6. 10.1126/science.1093686 14704433 PMC3244756

