# Supplementary material for: Characterization of bovine (Bos taurus) imprinted genes from genomic to amino acid attributes by data mining approaches
Source: PLoS One. 2019 Jun 6;14(6):e0217813. doi: 10.1371/journal.pone.0217813 (PMC6553745; doi:10.1371/journal.pone.0217813)
Supplement: S3 Table — (DOCX) [file pone.0217813.s003.docx]

S3 Table. The kappa value, accuracies, biallelic and imprint recalls and precisions of induction models computed on 10-fold CV.

| **Dataset** | **Models** | **Criteria** | **Kappa** | **Accuracy** | **Biallelic Recall** | **Imprint Recall** | **Biallelic Precision** | **Imprint Precision** |
| --- | --- | --- | --- | --- | --- | --- | --- | --- |
| **Chi Squared** | **Decision Tree** | **Accuracy** | **0.370** | **93.04** | **100.00** | **27.27** | **92.86** | **100.00** |
|  |  | **Gain Ratio** | **0.277** | **90.43** | **96.63** | **31.82** | **93.06** | **50.00** |
|  |  | **Gini Index** | **0.524** | **91.74** | **95.67** | **54.55** | **95.22** | **57.14** |
|  |  | **Info Gain** | **0.606** | **93.04** | **96.63** | **59.09** | **95.71** | **65.00** |
|  | **Random Tree** | **Accuracy** | **-0.006** | **90.00** | **99.52** | **0.00** | **90.39** | **0.00** |
|  |  | **Gain Ratio** | **0.275** | **92.17** | **99.04** | **27.27** | **92.79** | **75.00** |
|  |  | **Gini Index** | **0.484** | **91.74** | **96.63** | **45.45** | **94.37** | **58.82** |
|  |  | **Info Gain** | **0.407** | **90.87** | **95.67** | **45.45** | **94.31** | **52.63** |
|  | **Decision Stump** | **Accuracy** | **0.015** | **89.13** | **98.08** | **4.55** | **90.67** | **20.00** |
|  |  | **Gain Ratio** | **0.015** | **89.13** | **98.08** | **4.55** | **90.67** | **20.00** |
|  |  | **Gini Index** | **0.268** | **90.00** | **96.63** | **27.27** | **92.63** | **46.15** |
|  |  | **Info Gain** | **0.025** | **89.57** | **98.56** | **4.55** | **90.71** | **25.00** |
|  | **Random Forest** | **Accuracy** | **0.111** | **91.30** | **100.00** | **9.09** | **91.23** | **100.00** |
|  |  | **Gain Ratio** | **0.247** | **91.30** | **98.56** | **22.73** | **92.34** | **62.50** |
|  |  | **Gini Index** | **0.499** | **93.91** | **100.00** | **36.36** | **93.69** | **100.00** |
|  |  | **Info Gain** | **0.634** | **95.22** | **100.00** | **50.00** | **94.98** | **100.00** |

| **Dataset** | **Models** | **Criteria** | **Kappa** | **Accuracy** | **Biallelic Recall** | **Imprint Recall** | **Biallelic Precision** | **Imprint Precision** |
| --- | --- | --- | --- | --- | --- | --- | --- | --- |
| **Info Gain** | **Decision Tree** | **Accuracy** | **0.337** | **92.17** | **99.04** | **27.27** | **92.79** | **75.00** |
|  |  | **Gain Ratio** | **0.232** | **90.43** | **97.12** | **27.27** | **92.66** | **50.00** |
|  |  | **Gini Index** | **0.520** | **91.74** | **96.15** | **50.00** | **94.79** | **57.89** |
|  |  | **Info Gain** | **0.590** | **93.04** | **95.67** | **68.18** | **96.60** | **62.50** |
|  | **Random Tree** | **Accuracy** | **0.033** | **90.43** | **99.52** | **4.55** | **90.79** | **50.00** |
|  |  | **Gain Ratio** | **0.289** | **90.00** | **96.63** | **27.27** | **92.63** | **46.15** |
|  |  | **Gini Index** | **0.244** | **90.43** | **97.60** | **22.73** | **92.27** | **50.00** |
|  |  | **Info Gain** | **0.356** | **91.30** | **97.60** | **31.82** | **93.12** | **58.33** |
|  | **Decision Stump** | **Accuracy** | **0.021** | **89.57** | **98.56** | **4.55** | **90.71** | **25.00** |
|  |  | **Gain Ratio** | **0.021** | **89.57** | **98.56** | **4.55** | **90.71** | **25.00** |
|  |  | **Gini Index** | **0.268** | **90.00** | **96.63** | **27.27** | **92.63** | **46.15** |
|  |  | **Info Gain** | **0.025** | **89.57** | **98.56** | **4.55** | **90.71** | **0.25** |
|  | **Random Forest** | **Accuracy** | **0.058** | **90.43** | **99.52** | **4.55** | **90.79** | **50.00** |
|  |  | **Gain Ratio** | **0.351** | **93.04** | **100.00** | **27.27** | **92.86** | **100.00** |
|  |  | **Gini Index** | **0.483** | **94.35** | **100.00** | **40.91** | **94.12** | **100.00** |
|  |  | **Info Gain** | **0.515** | **94.35** | **99.52** | **45.45** | **94.52** | **90.91** |

| **Dataset** | **Models** | **Criteria** | **Kappa** | **Accuracy** | **Biallelic Recall** | **Imprint Recall** | **Biallelic Precision** | **Imprint Precision** |
| --- | --- | --- | --- | --- | --- | --- | --- | --- |
| **Deviation** | **Decision Tree** | **Accuracy** | **0.00** | **90.43** | **100.00** | **0.00** | **90.43** | **0.00** |
|  |  | **Gain Ratio** | **0.00** | **90.43** | **100.00** | **0.00** | **90.43** | **0.00** |
|  |  | **Gini Index** | **0.00** | **90.43** | **100.00** | **0.00** | **90.43** | **0.00** |
|  |  | **Info Gain** | **-0.019** | **88.70** | **98.08** | **0.00** | **90.27** | **0.00** |
|  | **Random Tree** | **Accuracy** | **0.00** | **90.43** | **100.00** | **0.00** | **90.43** | **0.00** |
|  |  | **Gain Ratio** | **0.00** | **90.43** | **100.00** | **0.00** | **90.43** | **0.00** |
|  |  | **Gini Index** | **0.149** | **85.65** | **91.83** | **27.27** | **92.27** | **26.09** |
|  |  | **Info Gain** | **-0.019** | **88.70** | **98.08** | **0.00** | **90.27** | **0.00** |
|  | **Decision Stump** | **Accuracy** | **0.00** | **90.43** | **100.00** | **0.00** | **90.43** | **0.00** |
|  |  | **Gain Ratio** | **0.00** | **90.43** | **100.00** | **0.00** | **90.43** | **0.00** |
|  |  | **Gini Index** | **0.00** | **90.43** | **100.00** | **0.00** | **90.43** | **0.00** |
|  |  | **Info Gain** | **0.00** | **90.43** | **100.00** | **0.00** | **90.43** | **0.00** |
|  | **Random Forest** | **Accuracy** | **0.00** | **90.43** | **100.00** | **0.00** | **90.43** | **0.00** |
|  |  | **Gain Ratio** | **0.00** | **90.43** | **100.00** | **0.00** | **90.43** | **0.00** |
|  |  | **Gini Index** | **0.00** | **90.43** | **100.00** | **0.00** | **90.43** | **0.00** |
|  |  | **Info Gain** | **0.017** | **88.70** | **97.60** | **4.55** | **90.62** | **16.67** |

| **Dataset** | **Models** | **Criteria** | **Kappa** | **Accuracy** | **Biallelic Recall** | **Imprint Recall** | **Biallelic Precision** | **Imprint Precision** |
| --- | --- | --- | --- | --- | --- | --- | --- | --- |
| **Gini Index** | **Decision Tree** | **Accuracy** | **0.143** | **89.57** | **97.12** | **18.18** | **91.82** | **40.00** |
|  |  | **Gain Ratio** | **0.100** | **88.70** | **96.63** | **13.64** | **91.36** | **30.00** |
|  |  | **Gini Index** | **0.347** | **88.26** | **93.27** | **40.91** | **93.72** | **39.13** |
|  |  | **Info Gain** | **0.367** | **88.70** | **93.75** | **40.91** | **93.75** | **40.91** |
|  | **Random Tree** | **Accuracy** | **-0.006** | **90.00** | **99.52** | **0.00** | **90.39** | **0.00** |
|  |  | **Gain Ratio** | **0.364** | **91.74** | **97.60** | **36.36** | **93.55** | **61.54** |
|  |  | **Gini Index** | **0.328** | **90.87** | **97.12** | **31.82** | **93.09** | **53.85** |
|  |  | **Info Gain** | **0.388** | **92.17** | **98.08** | **36.36** | **93.58** | **66.67** |
|  | **Decision Stump** | **Accuracy** | **-0.007** | **90.00** | **99.52** | **0.00** | **90.39** | **0.00** |
|  |  | **Gain Ratio** | **-0.007** | **90.00** | **99.52** | **0.00** | **90.39** | **0.00** |
|  |  | **Gini Index** | **0.268** | **90.00** | **96.63** | **27.27** | **92.63** | **46.15** |
|  |  | **Info Gain** | **0.025** | **89.57** | **98.56** | **4.55** | **90.71** | **25.00** |
|  | **Random Forest** | **Accuracy** | **0.111** | **91.30** | **100.00** | **9.09** | **91.23** | **100.00** |
|  |  | **Gain Ratio** | **0.172** | **89.57** | **97.12** | **18.18** | **91.82** | **40.00** |
|  |  | **Gini Index** | **0.344** | **92.17** | **98.56** | **31.82** | **93.18** | **70.00** |
|  |  | **Info Gain** | **0.395** | **92.61** | **99.04** | **31.82** | **93.21** | **77.78** |

| **Dataset** | **Models** | **Criteria** | **Kappa** | **Accuracy** | **Biallelic Recall** | **Imprint Recall** | **Biallelic Precision** | **Imprint Precision** |
| --- | --- | --- | --- | --- | --- | --- | --- | --- |
| **Info Gain Ratio** | **Decision Tree** | **Accuracy** | **0.073** | **87.39** | **95.19** | **13.64** | **91.24** | **23.08** |
|  |  | **Gain Ratio** | **-0.002** | **87.83** | **96.63** | **4.55** | **90.54** | **12.50** |
|  |  | **Gini Index** | **0.368** | **87.83** | **92.31** | **45.45** | **94.12** | **38.46** |
|  |  | **Info Gain** | **0.224** | **86.96** | **92.79** | **31.82** | **92.79** | **31.82** |
|  | **Random Tree** | **Accuracy** | **0.00** | **90.43** | **100.00** | **0.00** | **90.43** | **0.00** |
|  |  | **Gain Ratio** | **0.100** | **89.57** | **97.60** | **13.64** | **91.44** | **37.50** |
|  |  | **Gini Index** | **0.380** | **90.87** | **96.63** | **36.36** | **93.49** | **53.33** |
|  |  | **Info Gain** | **0.206** | **90.00** | **97.12** | **22.73** | **92.24** | **45.45** |
|  | **Decision Stump** | **Accuracy** | **0.020** | **89.57** | **98.56** | **4.55** | **90.71** | **25.00** |
|  |  | **Gain Ratio** | **0.020** | **89.57** | **98.56** | **4.55** | **90.71** | **25.00** |
|  |  | **Gini Index** | **0.268** | **90.00** | **96.63** | **27.27** | **29.63** | **46.15** |
|  |  | **Info Gain** | **0.070** | **89.57** | **98.08** | **9.09** | **91.07** | **33.33** |
|  | **Random Forest** | **Accuracy** | **0.110** | **90.87** | **99.52** | **9.09** | **91.19** | **66.67** |
|  |  | **Gain Ratio** | **0.258** | **92.17** | **100.00** | **18.18** | **92.04** | **100.00** |
|  |  | **Gini Index** | **0.395** | **92.61** | **99.04** | **31.82** | **93.21** | **77.78** |
|  |  | **Info Gain** | **0.315** | **92.17** | **99.04** | **27.27** | **92.79** | **75.00** |

| **Dataset** | **Models** | **Criteria** | **Kappa** | **Accuracy** | **Biallelic Recall** | **Imprint Recall** | **Biallelic Precision** | **Imprint Precision** |
| --- | --- | --- | --- | --- | --- | --- | --- | --- |
| **PCA** | **Decision Tree** | **Accuracy** | **0.00** | **90.43** | **100.00** | **0.00** | **90.43** | **0.00** |
|  |  | **Gain Ratio** | **0.00** | **90.43** | **100.00** | **0.00** | **90.43** | **0.00** |
|  |  | **Gini Index** | **0.00** | **90.43** | **100.00** | **0.00** | **90.43** | **0.00** |
|  |  | **Info Gain** | **-0.010** | **89.57** | **99.04** | **0.00** | **90.35** | **0.00** |
|  | **Random Tree** | **Accuracy** | **0.00** | **90.43** | **100.00** | **0.00** | **90.43** | **0.00** |
|  |  | **Gain Ratio** | **0.00** | **90.43** | **100.00** | **0.00** | **90.43** | **0.00** |
|  |  | **Gini Index** | **-0.019** | **88.70** | **98.08** | **0.00** | **90.27** | **0.00** |
|  |  | **Info Gain** | **-0.010** | **89.57** | **99.04** | **0.00** | **90.35** | **0.00** |
|  | **Decision Stump** | **Accuracy** | **0.00** | **90.43** | **100.00** | **0.00** | **90.43** | **0.00** |
|  |  | **Gain Ratio** | **0.00** | **90.43** | **100.00** | **0.00** | **90.43** | **0.00** |
|  |  | **Gini Index** | **0.00** | **90.43** | **100.00** | **0.00** | **90.43** | **0.00** |
|  |  | **Info Gain** | **0.00** | **90.43** | **100.00** | **0.00** | **90.43** | **0.00** |
|  | **Random Forest** | **Accuracy** | **0.00** | **90.43** | **100.00** | **0.00** | **90.43** | **0.00** |
|  |  | **Gain Ratio** | **0.00** | **90.43** | **100.00** | **0.00** | **90.43** | **0.00** |
|  |  | **Gini Index** | **0.018** | **89.13** | **98.08** | **4.55** | **90.67** | **20.00** |
|  |  | **Info Gain** | **-0.023** | **88.70** | **98.08** | **0.00** | **90.27** | **0.00** |

| **Dataset** | **Models** | **Criteria** | **Kappa** | **Accuracy** | **Biallelic Recall** | **Imprint Recall** | **Biallelic Precision** | **Imprint Precision** |
| --- | --- | --- | --- | --- | --- | --- | --- | --- |
| **Correlation** | **Decision Tree** | **Accuracy** | **0.092** | **88.70** | **96.63** | **13.64** | **91.36** | **30.00** |
|  |  | **Gain Ratio** | **0.068** | **88.26** | **96.63** | **9.09** | **90.95** | **22.22** |
|  |  | **Gini Index** | **0.307** | **87.83** | **92.79** | **40.91** | **93.69** | **37.50** |
|  |  | **Info Gain** | **0.474** | **91.74** | **96.15** | **50.00** | **94.79** | **57.89** |
|  | **Random Tree** | **Accuracy** | **-0.006** | **90.00** | **99.52** | **0.00** | **90.39** | **0.00** |
|  |  | **Gain Ratio** | **0.176** | **91.74** | **100.00** | **13.64** | **91.63** | **100.00** |
|  |  | **Gini Index** | **0.389** | **93.04** | **99.04** | **36.36** | **93.64** | **80.00** |
|  |  | **Info Gain** | **0.322** | **91.74** | **98.08** | **31.82** | **93.15** | **63.64** |
|  | **Decision Stump** | **Accuracy** | **0.091** | **90.43** | **99.04** | **9.09** | **91.15** | **50.00** |
|  |  | **Gain Ratio** | **0.091** | **90.43** | **99.04** | **9.09** | **91.15** | **50.00** |
|  |  | **Gini Index** | **0.268** | **90.00** | **96.63** | **27.27** | **92.63** | **46.15** |
|  |  | **Info Gain** | **0.025** | **89.57** | **98.56** | **4.55** | **90.71** | **25.00** |
|  | **Random Forest** | **Accuracy** | **0.033** | **90.00** | **99.04** | **4.55** | **90.75** | **33.33** |
|  |  | **Gain Ratio** | **0.084** | **90.00** | **98.56** | **9.09** | **91.11** | **40.00** |
|  |  | **Gini Index** | **0.150** | **90.87** | **99.04** | **13.64** | **91.56** | **60.00** |
|  |  | **Info Gain** | **0.351** | **93.04** | **100.00** | **27.27** | **92.86** | **100.00** |

| **Dataset** | **Models** | **Criteria** | **Kappa** | **Accuracy** | **Biallelic Recall** | **Imprint Recall** | **Biallelic Precision** | **Imprint Precision** |
| --- | --- | --- | --- | --- | --- | --- | --- | --- |
| **Relief** | **Decision Tree** | **Accuracy** | **0.266** | **91.30** | **98.56** | **22.73** | **92.34** | **62.50** |
|  |  | **Gain Ratio** | **0.119** | **89.13** | **97.12** | **13.64** | **91.40** | **33.33** |
|  |  | **Gini Index** | **0.420** | **91.74** | **97.12** | **40.91** | **93.95** | **60.00** |
|  |  | **Info Gain** | **0.470** | **90.87** | **94.71** | **54.55** | **95.17** | **52.17** |
|  | **Random Tree** | **Accuracy** | **-0.012** | **89.57** | **99.04** | **0.00** | **90.35** | **0.00** |
|  |  | **Gain Ratio** | **0.00** | **90.43** | **100.00** | **0.00** | **90.43** | **0.00** |
|  |  | **Gini Index** | **0.144** | **89.57** | **97.12** | **18.18** | **91.82** | **40.00** |
|  |  | **Info Gain** | **0.144** | **91.30** | **99.52** | **13.64** | **91.59** | **75.00** |
|  | **Decision Stump** | **Accuracy** | **-0.019** | **89.13** | **98.56** | **0.00** | **90.31** | **0.00** |
|  |  | **Gain Ratio** | **-0.019** | **89.13** | **98.56** | **0.00** | **90.31** | **0.00** |
|  |  | **Gini Index** | **0.268** | **90.00** | **96.63** | **27.27** | **92.63** | **46.15** |
|  |  | **Info Gain** | **0.070** | **89.57** | **98.08** | **0.09** | **91.07** | **0.33** |
|  | **Random Forest** | **Accuracy** | **0.00** | **90.43** | **100.00** | **0.00** | **90.43** | **0.00** |
|  |  | **Gain Ratio** | **0.181** | **90.87** | **99.04** | **13.64** | **91.56** | **60.00** |
|  |  | **Gini Index** | **0.272** | **91.74** | **99.04** | **22.73** | **92.38** | **71.43** |
|  |  | **Info Gain** | **0.338** | **92.61** | **99.52** | **27.27** | **92.83** | **85.71** |

| **Dataset** | **Models** | **Criteria** | **Kappa** | **Accuracy** | **Biallelic Recall** | **Imprint Recall** | **Biallelic Precision** | **Imprint Precision** |
| --- | --- | --- | --- | --- | --- | --- | --- | --- |
| **Rule** | **Decision Tree** | **Accuracy** | **0.157** | **89.13** | **96.63** | **18.18** | **91.78** | **36.36** |
|  |  | **Gain Ratio** | **0.104** | **89.13** | **97.12** | **13.64** | **91.40** | **33.33** |
|  |  | **Gini Index** | **0.292** | **89.13** | **95.19** | **31.82** | **92.96** | **41.18** |
|  |  | **Info Gain** | **0.293** | **87.83** | **93.27** | **36.36** | **93.27** | **36.36** |
|  | **Random Tree** | **Accuracy** | **0.00** | **90.43** | **100.00** | **0.00** | **90.43** | **0.00** |
|  |  | **Gain Ratio** | **0.00** | **90.43** | **100.00** | **0.00** | **90.43** | **0.00** |
|  |  | **Gini Index** | **0.257** | **90.87** | **98.08** | **22.73** | **92.31** | **55.56** |
|  |  | **Info Gain** | **0.305** | **92.61** | **100.00** | **22.73** | **92.44** | **100.00** |
|  | **Decision Stump** | **Accuracy** | **-0.007** | **90.00** | **99.52** | **0.00** | **90.39** | **0.00** |
|  |  | **Gain Ratio** | **-0.007** | **90.00** | **99.52** | **0.00** | **90.39** | **0.00** |
|  |  | **Gini Index** | **0.268** | **90.00** | **96.63** | **27.27** | **92.63** | **46.15** |
|  |  | **Info Gain** | **0.070** | **89.57** | **98.08** | **9.09** | **91.07** | **33.33** |
|  | **Random Forest** | **Accuracy** | **0.111** | **91.30** | **100.00** | **9.09** | **91.23** | **100.00** |
|  |  | **Gain Ratio** | **0.143** | **90.87** | **99.04** | **13.64** | **91.56** | **60.00** |
|  |  | **Gini Index** | **0.340** | **93.04** | **100.00** | **27.27** | **92.86** | **100.00** |
|  |  | **Info Gain** | **0.383** | **93.04** | **99.52** | **31.82** | **93.24** | **87.50** |

| **Dataset** | **Models** | **Criteria** | **Kappa** | **Accuracy** | **Biallelic Recall** | **Imprint Recall** | **Biallelic Precision** | **Imprint Precision** |
| --- | --- | --- | --- | --- | --- | --- | --- | --- |
| **Uncertainty** | **Decision Tree** | **Accuracy** | **0.239** | **90.43** | **97.60** | **22.73** | **92.27** | **50.00** |
|  |  | **Gain Ratio** | **0.344** | **92.17** | **99.04** | **27.27** | **92.79** | **75.00** |
|  |  | **Gini Index** | **0.354** | **90.43** | **96.15** | **36.36** | **93.46** | **50.00** |
|  |  | **Info Gain** | **0.354** | **90.43** | **96.15** | **36.36** | **93.46** | **50.00** |
|  | **Random Tree** | **Accuracy** | **-0.006** | **90.00** | **99.52** | **0.00** | **90.39** | **0.000** |
|  |  | **Gain Ratio** | **0.065** | **90.87** | **100.00** | **4.55** | **90.83** | **100.00** |
|  |  | **Gini Index** | **0.312** | **91.30** | **98.08** | **27.27** | **92.73** | **60.00** |
|  |  | **Info Gain** | **0.343** | **92.17** | **99.04** | **27.27** | **92.79** | **75.00** |
|  | **Decision Stump** | **Accuracy** | **-0.006** | **90.00** | **99.52** | **0.00** | **90.39** | **0.00** |
|  |  | **Gain Ratio** | **-0.006** | **90.00** | **99.52** | **0.00** | **90.39** | **0.00** |
|  |  | **Gini Index** | **0.451** | **91.74** | **96.15** | **50.00** | **94.79** | **57.89** |
|  |  | **Info Gain** | **0.451** | **91.74** | **96.15** | **50.00** | **94.79** | **57.89** |
|  | **Random Forest** | **Accuracy** | **-0.018** | **88.70** | **98.08** | **0.00** | **90.27** | **0.00** |
|  |  | **Gain Ratio** | **0.016** | **88.26** | **97.12** | **4.55** | **90.58** | **14.29** |
|  |  | **Gini Index** | **0.248** | **90.00** | **97.12** | **22.73** | **92.24** | **45.45** |
|  |  | **Info Gain** | **0.267** | **90.43** | **97.60** | **22.73** | **92.27** | **50.00** |

| **Dataset** | **Models** | **Criteria** | **Kappa** | **Accuracy** | **Biallelic Recall** | **Imprint Recall** | **Biallelic Precision** | **Imprint Precision** |
| --- | --- | --- | --- | --- | --- | --- | --- | --- |
| **SVM** | **Decision Tree** | **Accuracy** | **0.321** | **92.61** | **99.04** | **31.82** | **93.21** | **77.78** |
|  |  | **Gain Ratio** | **0.429** | **91.74** | **96.63** | **45.45** | **94.37** | **58.82** |
|  |  | **Gini Index** | **0.416** | **91.74** | **97.12** | **40.91** | **93.95** | **60.00** |
|  |  | **Info Gain** | **0.419** | **91.74** | **96.63** | **45.45** | **94.37** | **58.82** |
|  | **Random Tree** | **Accuracy** | **0.000** | **90.43** | **100.00** | **0.00** | **90.43** | **0.00** |
|  |  | **Gain Ratio** | **0.302** | **92.17** | **99.04** | **27.27** | **92.79** | **75.00** |
|  |  | **Gini Index** | **0.507** | **92.61** | **97.12** | **50.00** | **94.84** | **64.71** |
|  |  | **Info Gain** | **0.439** | **92.17** | **97.60** | **40.91** | **93.98** | **64.29** |
|  | **Decision Stump** | **Accuracy** | **0.065** | **90.87** | **100.00** | **4.55** | **90.83** | **100.00** |
|  |  | **Gain Ratio** | **0.065** | **90.87** | **100.00** | **4.55** | **90.83** | **100.00** |
|  |  | **Gini Index** | **0.268** | **90.00** | **96.63** | **27.27** | **92.63** | **46.15** |
|  |  | **Info Gain** | **0.070** | **89.57** | **98.08** | **9.09** | **91.07** | **33.33** |
|  | **Random Forest** | **Accuracy** | **0.047** | **90.87** | **100.00** | **4.55** | **90.83** | **100.00** |
|  |  | **Gain Ratio** | **0.339** | **92.61** | **99.52** | **27.27** | **92.83** | **85.71** |
|  |  | **Gini Index** | **0.322** | **93.04** | **100.00** | **27.27** | **92.86** | **100.00** |
|  |  | **Info Gain** | **0.516** | **94.35** | **100.00** | **40.91** | **94.12** | **100.00** |

| **Dataset** | **Models** | **Criteria** | **Kappa** | **Accuracy** | **Biallelic Recall** | **Imprint Recall** | **Biallelic Precision** | **Imprint Precision** |
| --- | --- | --- | --- | --- | --- | --- | --- | --- |
| **Mds** | **Decision Tree** | **Accuracy** | **0.156** | **88.26** | **95.67** | **18.18** | **91.71** | **30.77** |
|  |  | **Gain Ratio** | **-0.002** | **87.83** | **96.63** | **4.55** | **90.54** | **12.50** |
|  |  | **Gini Index** | **0.328** | **90.00** | **96.15** | **31.82** | **93.02** | **46.67** |
|  |  | **Info Gain** | **0.488** | **91.74** | **94.71** | **63.64** | **96.10** | **56.00** |
|  | **Random Tree** | **Accuracy** | **0.00** | **90.43** | **100.00** | **0.00** | **90.43** | **0.00** |
|  |  | **Gain Ratio** | **0.053** | **89.57** | **98.56** | **4.55** | **90.71** | **25.00** |
|  |  | **Gini Index** | **-0.019** | **88.70** | **98.08** | **0.00** | **90.27** | **0.00** |
|  |  | **Info Gain** | **0.051** | **90.00** | **99.04** | **4.55** | **90.75** | **33.33** |
|  | **Decision Stump** | **Accuracy** | **0.020** | **89.57** | **98.56** | **4.55** | **90.71** | **25.00** |
|  |  | **Gain Ratio** | **0.020** | **89.57** | **98.56** | **4.55** | **90.71** | **25.00** |
|  |  | **Gini Index** | **0.268** | **90.00** | **96.63** | **27.27** | **92.63** | **46.15** |
|  |  | **Info Gain** | **0.025** | **89.57** | **98.56** | **4.55** | **90.71** | **0.25** |
|  | **Random Forest** | **Accuracy** | **0.00** | **90.43** | **100.00** | **0.00** | **90.43** | **0.00** |
|  |  | **Gain Ratio** | **0.00** | **90.43** | **100.00** | **0.00** | **90.43** | **0.00** |
|  |  | **Gini Index** | **0.176** | **91.74** | **100.00** | **13.64** | **91.63** | **100.00** |
|  |  | **Info Gain** | **0.340** | **93.04** | **100.00** | **27.27** | **92.86** | **100.00** |
