# Supplementary material for: Characterization of bovine (Bos taurus) imprinted genes from genomic to amino acid attributes by data mining approaches
Source: PLoS One. 2019 Jun 6;14(6):e0217813. doi: 10.1371/journal.pone.0217813 (PMC6553745; doi:10.1371/journal.pone.0217813)
Supplement: S4 Table — (DOCX) [file pone.0217813.s004.docx]

S4 Table. The kappa value, accuracies, biallelic and imprint recalls and precisions of neural network and Bayesian models computed on 10-fold CV.

| **Model** | **Dataset** | **Kappa** | **Accuracy** | **Biallelic Recall** | **Imprint Recall** | **Biallelic Precision** | **Imprint Precision** |
| --- | --- | --- | --- | --- | --- | --- | --- |
| **Deep Learning** | **Chi Squared** | **0.440** | **90.82** | **95.54** | **45.20** | **94.38** | **52.30** |
|  | **Info Gain** | **0.609** | **93.51** | **96.88** | **61.67** | **95.98** | **67.62** |
|  | **Deviation** | **0.096** | **59.57** | **58.90** | **65.89** | **94.23** | **14.50** |
|  | **Gini Index** | **0.396** | **89.81** | **94.65** | **44.01** | **94.11** | **46.53** |
|  | **Info Gain Ratio** | **0.287** | **88.59** | **94.55** | **32.19** | **92.95** | **38.46** |
|  | **PCA** | **0.143** | **63.21** | **62.16** | **73.05** | **95.62** | **16.96** |
|  | **Correlation** | **0.437** | **91.06** | **96.00** | **44.34** | **94.22** | **53.99** |
|  | **Relief** | **0.278** | **88.77** | **94.98** | **30.12** | **92.78** | **38.81** |
|  | **Rule** | **0.280** | **88.34** | **94.29** | **32.10** | **92.92** | **37.29** |
|  | **Uncertainty** | **0.302** | **87.15** | **92.12** | **40.13** | **93.57** | **35.02** |
|  | **SVM** | **0.676** | **94.60** | **97.43** | **67.90** | **96.63** | **73.63** |
|  | **Mds** | **0.282** | **91.67** | **99.26** | **19.95** | **92.14** | **73.93** |

| **Model** | **Dataset** | **Kappa** | **Accuracy** | **Biallelic Recall** | **Imprint Recall** | **Biallelic Precision** | **Imprint Precision** |
| --- | --- | --- | --- | --- | --- | --- | --- |
| **AutoMPL** | **Chi Squared** | **0.471** | **90.43** | **94.71** | **50.00** | **94.71** | **50.00** |
|  | **Info Gain** | **0.645** | **94.35** | **97.12** | **68.18** | **96.65** | **71.43** |
|  | **Deviation** | **0.00** | **90.43** | **100.00** | **0.00** | **90.43** | **0.00** |
|  | **Gini Index** | **0.451** | **90.87** | **95.67** | **45.45** | **94.31** | **52.63** |
|  | **Info Gain Ratio** | **0.352** | **90.00** | **95.67** | **36.36** | **93.43** | **47.06** |
|  | **PCA** | **0.00** | **90.43** | **100.00** | **0.00** | **90.43** | **0.00** |
|  | **Correlation** | **0.476** | **91.74** | **95.67** | **54.55** | **95.22** | **57.14** |
|  | **Relief** | **0.247** | **90.00** | **97.12** | **22.73** | **92.24** | **45.45** |
|  | **Rule** | **0.228** | **91.30** | **99.04** | **18.18** | **91.96** | **66.67** |
|  | **Uncertainty** | **0.162** | **90.43** | **98.56** | **13.64** | **91.52** | **50.00** |
|  | **SVM** | **0.628** | **95.22** | **99.04** | **59.09** | **95.81** | **86.67** |
|  | **Mds** | **0.439** | **91.74** | **96.63** | **45.45** | **94.37** | **58.82** |

| **Model** | **Dataset** | **Kappa** | **Accuracy** | **Biallelic Recall** | **Imprint Recall** | **Biallelic Precision** | **Imprint Precision** |
| --- | --- | --- | --- | --- | --- | --- | --- |
| **Neural Net** | **Chi Squared** | **0.475** | **90.00** | **94.23** | **50.00** | **94.69** | **47.83** |
|  | **Info Gain** | **0.586** | **93.04** | **95.67** | **68.18** | **96.60** | **62.50** |
|  | **Deviation** | **0.00** | **90.43** | **100.00** | **0.00** | **90.43** | **0.00** |
|  | **Gini Index** | **0.428** | **90.87** | **95.67** | **45.45** | **94.31** | **52.63** |
|  | **Info Gain Ratio** | **0.409** | **90.00** | **94.71** | **45.45** | **94.26** | **47.62** |
|  | **PCA** | **0.00** | **90.43** | **100.00** | **0.00** | **90.43** | **0.00** |
|  | **Correlation** | **0.467** | **90.00** | **93.75** | **54.55** | **95.12** | **48.00** |
|  | **Relief** | **0.255** | **88.26** | **94.71** | **27.27** | **92.49** | **35.29** |
|  | **Rule** | **0.224** | **89.13** | **95.19** | **31.82** | **92.96** | **41.18** |
|  | **Uncertainty** | **0.133** | **88.70** | **96.15** | **18.18** | **91.74** | **33.33** |
|  | **SVM** | **0.651** | **94.35** | **97.12** | **68.18** | **96.65** | **71.43** |
|  | **Mds** | **0.509** | **91.30** | **95.19** | **54.55** | **95.19** | **54.55** |

| **Model** | **Dataset** | **Kappa** | **Accuracy** | **Biallelic Recall** | **Imprint Recall** | **Biallelic Precision** | **Imprint Precision** |
| --- | --- | --- | --- | --- | --- | --- | --- |
| **Perceptron** | **Chi Squared** | **0.382** | **81.30** | **81.73** | **77.27** | **97.14** | **30.91** |
|  | **Info Gain** | **0.633** | **93.91** | **95.67** | **77.27** | **97.55** | **65.38** |
|  | **Deviation** | **0.050** | **44.78** | **41.83** | **72.73** | **93.55** | **11.68** |
|  | **Gini Index** | **0.412** | **83.48** | **84.13** | **77.27** | **97.22** | **34.00** |
|  | **Info Gain Ratio** | **0.375** | **87.83** | **91.83** | **50.00** | **94.55** | **39.29** |
|  | **PCA** | **0.029** | **29.13** | **22.60** | **90.91** | **95.92** | **11.05** |
|  | **Correlation** | **0.496** | **90.00** | **93.27** | **59.09** | **95.57** | **48.15** |
|  | **Relief** | **0.427** | **88.26** | **91.35** | **59.09** | **95.48** | **41.94** |
|  | **Rule** | **0.189** | **69.57** | **69.71** | **68.18** | **95.39** | **19.23** |
|  | **Uncertainty** | **0.106** | **42.61** | **37.02** | **95.45** | **98.72** | **13.82** |
|  | **SVM** | **0.610** | **90.43** | **90.38** | **90.91** | **98.95** | **50.00** |
|  | **Mds** | **0.585** | **92.61** | **95.67** | **63.64** | **96.14** | **60.87** |

| **Model** | **Dataset** | **Kappa** | **Accuracy** | **Biallelic Recall** | **Imprint Recall** | **Biallelic Precision** | **Imprint Precision** |
| --- | --- | --- | --- | --- | --- | --- | --- |
| **Naive Bayse** | **Chi Squared** | **0.317** | **89.13** | **94.23** | **40.91** | **93.78** | **42.86** |
|  | **Info Gain** | **0.549** | **91.74** | **94.23** | **68.18** | **96.55** | **55.56** |
|  | **Deviation** | **0.165** | **61.30** | **59.13** | **81.82** | **96.85** | **17.48** |
|  | **Gini Index** | **0.339** | **88.26** | **92.79** | **45.45** | **94.15** | **40.00** |
|  | **Info Gain Ratio** | **0.246** | **86.96** | **92.31** | **36.36** | **93.20** | **33.33** |
|  | **PCA** | **0.159** | **63.48** | **62.02** | **77.27** | **96.27** | **17.71** |
|  | **Correlation** | **0.334** | **88.26** | **92.79** | **45.45** | **94.15** | **40.00** |
|  | **Relief** | **0.310** | **83.04** | **86.54** | **50.00** | **94.24** | **28.21** |
|  | **Rule** | **0.207** | **86.52** | **90.31** | **31.82** | **92.75** | **30.43** |
|  | **Uncertainty** | **0.225** | **90.00** | **97.12** | **22.73** | **92.24** | **45.45** |
|  | **SVM** | **0.657** | **93.48** | **95.19** | **77.27** | **97.54** | **62.96** |
|  | **Mds** | **0.463** | **87.83** | **89.42** | **72.73** | **96.88** | **42.11** |

| **Model** | **Dataset** | **Kappa** | **Accuracy** | **Biallelic Recall** | **Imprint Recall** | **Biallelic Precision** | **Imprint Precision** |
| --- | --- | --- | --- | --- | --- | --- | --- |
| **Naive Bayes (Kernel)** | **Chi Squared** | **0.361** | **91.74** | **97.60** | **36.36** | **93.55** | **61.54** |
|  | **Info Gain** | **0.754** | **96.09** | **98.08** | **77.27** | **97.61** | **80.95** |
|  | **Deviation** | **0.135** | **60.00** | **58.17** | **77.27** | **96.03** | **16.35** |
|  | **Gini Index** | **0.311** | **90.87** | **96.63** | **36.36** | **93.49** | **53.33** |
|  | **Info Gain Ratio** | **0.235** | **90.00** | **96.63** | **27.27** | **92.63** | **46.15** |
|  | **PCA** | **0.125** | **59.13** | **57.21** | **77.27** | **95.97** | **16.04** |
|  | **Correlation** | **0.372** | **91.74** | **97.12** | **40.91** | **93.95** | **60.00** |
|  | **Relief** | **0.317** | **88.26** | **93.75** | **36.36** | **93.30** | **38.10** |
|  | **Rule** | **0.232** | **90.87** | **97.60** | **27.27** | **92.69** | **54.55** |
|  | **Uncertainty** | **0.275** | **91.74** | **98.56** | **27.27** | **92.76** | **66.67** |
|  | **SVM** | **0.638** | **94.78** | **98.08** | **63.64** | **96.23** | **77.78** |
|  | **Mds** | **0.00** | **90.43** | **100.00** | **0.00** | **90.43** | **0.00** |

| **Model** | **Dataset** | **Kappa** | **Accuracy** | **Biallelic Recall** | **Imprint Recall** | **Biallelic Precision** | **Imprint Precision** |
| --- | --- | --- | --- | --- | --- | --- | --- |
| **W-BayesNet** | **Chi Squared** | **0.616** | **93.48** | **96.63** | **63.64** | **96.17** | **66.67** |
|  | **Info Gain** | **0.690** | **95.22** | **98.08** | **68.18** | **96.68** | **78.95** |
|  | **Deviation** | **0.00** | **90.43** | **100.00** | **0.00** | **90.43** | **0.00** |
|  | **Gini Index** | **0.606** | **92.61** | **95.19** | **68.18** | **96.59** | **60.00** |
|  | **Info Gain Ratio** | **0.577** | **93.91** | **97.60** | **59.09** | **95.75** | **72.22** |
|  | **PCA** | **0.00** | **90.43** | **100.00** | **0.00** | **90.43** | **0.00** |
|  | **Correlation** | **0.643** | **94.35** | **97.60** | **63.64** | **96.21** | **73.68** |
|  | **Relief** | **0.679** | **95.22** | **98.56** | **63.64** | **96.24** | **82.35** |
|  | **Rule** | **0.539** | **92.61** | **96.63** | **54.55** | **95.26** | **63.16** |
|  | **Uncertainty** | **0.301** | **90.00** | **95.67** | **36.36** | **93.43** | **47.06** |
|  | **SVM** | **0.763** | **96.52** | **99.04** | **72.73** | **97.17** | **88.89** |
|  | **Mds** | **0.690** | **95.22** | **98.08** | **68.18** | **96.68** | **78.95** |
